# Oxygen tolerance and detoxification mechanisms of highly enriched planktonic anaerobic ammonium-oxidizing (anammox) bacteria

**DOI:** 10.1038/s43705-023-00251-7

**Published:** 2023-05-03

**Authors:** Satoshi Okabe, Shaoyu Ye, Xi Lan, Keishi Nukada, Haozhe Zhang, Kanae Kobayashi, Mamoru Oshiki

**Affiliations:** 1 Department of Environmental Engineering, Faculty of Engineering, Hokkaido University, North-13, West-8, Kita-ku, Sapporo, Hokkaido 060-8628 Japan; 2 Super-cutting-edge Grand and Advanced Research (SUGAR) Program, Japan Agency for Marine-Earth Science and Technology (JAMSTEC), 2-15 Natsushima-cho, Yokosuka, Kanagawa 237-0061 Japan

**Keywords:** Water microbiology, Physiology

## Abstract

Oxygen is a key regulatory factor of anaerobic ammonium oxidation (anammox). Although the inhibitory effect of oxygen is evident, a wide range of oxygen sensitivities of anammox bacteria have been reported so far, which makes it difficult to model the marine nitrogen loss and design anammox-based technologies. Here, oxygen tolerance and detoxification mechanisms of four genera of anammox bacteria; one marine species (“*Ca*. Scalindua sp.”) and four freshwater anammox species (“*Ca*. Brocadia sinica”, “*Ca*. Brocadia sapporoensis”, “*Ca*. Jettenia caeni”, and “*Ca*. Kuenenia stuttgartiensis”) were determined and then related to the activities of anti-oxidative enzymes. Highly enriched planktonic anammox cells were exposed to various levels of oxygen, and oxygen inhibition kinetics (50% inhibitory concentration (IC_50_) and upper O_2_ limits (DO_max_) of anammox activity) were quantitatively determined. A marine anammox species, “*Ca*. Scalindua sp.”, exhibited much higher oxygen tolerance capability (IC_50_ = 18.0 µM and DO_max_ = 51.6 µM) than freshwater species (IC_50_ = 2.7–4.2 µM and DO_max_ = 10.9–26.6 µM). The upper DO limit of “*Ca*. Scalindua sp.” was much higher than the values reported so far (~20 µM). Furthermore, the oxygen inhibition was reversible even after exposed to ambient air for 12–24 h. The comparative genome analysis confirmed that all anammox species commonly possess the genes considered to function for reduction of O_2_, superoxide anion (O_2_^•-^), and H_2_O_2_. However, the superoxide reductase (Sor)-peroxidase dependent detoxification system alone may not be sufficient for cell survival under microaerobic conditions. Despite the fact that anaerobes normally possess no or little superoxide dismutase (Sod) or catalase (Cat), only *Scalindua* exhibited high Sod activity of 22.6 ± 1.9 U/mg-protein with moderate Cat activity of 1.6 ± 0.7 U/mg-protein, which was consistent with the genome sequence analysis. This Sod-Cat dependent detoxification system could be responsible for the higher O_2_ tolerance of *Scalindua* than other freshwater anammox species lacking the Sod activity.

## Introduction

Oxygen plays a key role in regulating the marine nitrogen cycle even in oxygen minimum zones (OMZs). The OMZs constitute only 0.1% of total ocean volume (if the OMZ is defined as O_2_≦5 µM) but account for 20–40% of the oceanic nitrogen loss through denitrification and anaerobic ammonium oxidation (anammox) [1]. Such nitrogen loss (i.e., N_2_ production) intensively occurs at the oxic and anoxic interface of the OMZs [2, 3]. This indicates the importance of close microbial interactions among aerobic nitrification, denitrification, and anammox (anaerobically converts NH_4_^+^ to N_2_ with NO_2_^−^). The effects of oxygen levels on these processes are of central importance for understanding their contributions to the marine nitrogen cycle. Especially, the estimated contribution of anammox may vary greatly depending on the oxygen tolerance capability of marine anammox bacteria which basically determines the OMZ water volume with active anammox activity [4, 5]. Thus, their oxygen sensitivity and dynamic response to oxygen exposure need to be experimentally quantified.

A wide range of oxygen sensitivities of anammox bacteria have been reported for freshwater anammox species, whereas no data were available for marine species “*Ca*. Scalindua” so far [6, 7]. The previous O_2_ amendment studies using oceanic OMZ samples have shown that anammox activities were 50% inhibited at 0.9–16 µM [4, 8–11]. Similar or more severe oxygen sensitivity could be found for some freshwater anammox species like *B. anammoxidans*, *Brocadia sp*. and *K. stuttgartiensis*, demonstrating that anammox activities were completely inhibited at low dissolved oxygen (DO) levels (1.25–3.75 µM) [6, 12–14]. However, the anammox activities could be gradually recovered after oxygen inhibition, depending on the perceived inhibition levels [12–14].

A reported wide range of oxygen sensitivity could be attributed to many factors such as anammox species, degree of enrichment, form of biomass (planktonic or aggregated biomass), and measurement methods of anammox activity (e.g., NH_4_^+^ and/or NO_2_^−^ consumption or ^14+15^N_2_ gas production). In order to assess the reported aerotolerant natures more prudently, the anammox biomass used in those studies need to be clearly specified in detail since oxygen could be consumed by coexisting aerobes [4, 6, 13, 15] or aerobes shield anammox consortia from oxygen exposure in microbial aggregates, biofilms, or marine snow, both of which could result in overestimation of oxygen tolerance [16]. Therefore, aggregated or flocculated biomass often exhibited higher oxygen tolerance than planktonic biomass [17, 18]. In addition, there could be truly inter-species differences in oxygen sensitivity as suggested earlier by Yan et al. [19], which, however, has not been experimentally confirmed yet. Inherent oxygen inhibition kinetics (e.g., 50% inhibitory concentration (IC_50_) and upper O_2_ limits (DO_max_)) and recovery from O_2_ inhibition of anammox bacteria have not been directly determined using well-defined laboratory enrichment cultures to date.

Furthermore, oxygen detoxification mechanisms are largely unknown for anammox bacteria. It is generally known that when molecular oxygen (O_2_) diffuses into cells, reactive oxygen species (ROSs), such as superoxide anion (O_2_ ∙ ^-^) and hydrogen peroxide (H_2_O_2_) are generated as oxygen reduction by-products. Furthermore, O_2_ ∙ ^-^ can react with H_2_O_2_ to generate hydroxyl radicals (∙OH) in cells, which are the most potent oxidants among ROSs and thus cause the damage on DNA and proteins. Thus, organisms need to detoxify these ROSs by anti-oxidative enzymes such as superoxide dismutase (Sod), catalase (Cat), and peroxidases to survive in the presence of oxygen [20]. However, it is widely known that strict anaerobes usually do not possess Sod and/or Cat because these enzymes generate O_2_, propagating the further production of ROS [21]. Since anammox bacteria are regarded as obligate anaerobic bacteria [22], it is vital to investigate their ROS detoxification mechanism if they possess. It was speculated that “*Ca*. Brocadia sp.” most likely utilize a Sod-cytochrome *c* peroxidase system to detoxify ROS based on metagenomic and metatranscriptomic analyses [23]. However, abundance of “*Ca*. Brocadia sp.” in the biomass used in their study was relatively low (< 50%), and the activities of anti-oxidative enzymes have never been experimentally determined.

In the present study, oxygen tolerance capability of one marine species (“*Ca*. Scalindua sp.”) and four freshwater anammox species (“*Ca*. Brocadia sinica”, “*Ca*. Brocadia sapporoensis”, “*Ca*. Jettenia caeni”, and “*Ca*. Kuenenia stuttgartiensis”) were determined and then related to the activities of anti-oxidative enzymes (Sod, Cat, and peroxides). Free-living planktonic enrichment anammox cultures were further purified (> 99.8%) by applying Percoll density gradient centrifugation [24] and then subjected to these studies. The experimental results revealed that a marine anammox species, “*Ca*. Scalindua sp.”, exhibited the highest oxygen tolerance, which is likely attributed to the higher Sod activity. To the best of our knowledge, this is the first time to quantitatively verify that marine anammox bacteria possess higher oxygen tolerance than freshwater species and its oxygen detoxification mechanisms.

## Materials and methods

### Anammox biomass

Free-living planktonic cells of “*Ca*. Brocadia sinica”, “*Ca*. Brocadia sapporoensis”, “*Ca*. Jettenia caeni”, “*Ca*. Kuenenia stuttgartiensis” and “*Ca*. Scalindua sp.” were obtained from respective stock membrane bioreactors (MBRs) that have been maintained in our lab [7, 25–29]. The MBRs of “*Ca*. B. sapporoensis”, “*Ca*. Scalindua sp.” and “*Ca*. K. stuttgartiensis” were maintained at 25 °C, while the MBRs of “*Ca*. B. sinica” and “*Ca*. J. caeni” were maintained at 37 °C. The average purities of these MBR anammox cultures were 95 ± 3% of total bacteria, which were determined by FISH and q-PCR analysis [29–31]. Based on the measurements of 16 S rRNA gene copy numbers by qPCR, “*Ca*. B. sinica”, “*Ca*. K. stuttgartiensis”, “*Ca*. B. sapporoensis”, “*Ca*. J. caeni” and “*Ca*. Scalindua sp.” accounted for more than 99% of the anammox population in respective anammox MBR cultures. The MBR biomass was continuously stirred with magnetic stirrer to make sure the cells are planktonic state. The growth medium was made anoxically by flushing nitrogen gas (>99.995%) for at least 3 h, and an inert gas mixture (95% Argon and 5% CO_2_) was continuously bubbled into the bottom of the MBRs in order to maintain anaerobic conditions. The feeding medium contained the following compositions (mg L^−1^): FeSO_4_·7H_2_O (9.0), EDTA·4Na (5.0), NaCl (1.0), KCl (1.4), CaCl_2_·2H_2_O (1.4), MgSO_4_·7H_2_O (1.0), NaHCO_3_ (84.0), KH_2_PO_4_ (54.0), and trace element solution I and II (0.5 mL each) for all five MBRs [32]. For “*Ca*. Scalindua sp.” and “*Ca*. K. stuttgartiensis” cultures, the salinity of the medium was adjusted to 2.5% and 0.5% respectively by addition of an artificial sea salt (SEALIFE, Marine Tech, Tokyo, Japan). The pH of MBR cultures were in the range 7.3 to 7.5 without any pH adjustment. The nitrogen loading rate (NLR) was carefully controlled to avoid nitrite inhibition.

### Biomass preparation

One night before the start-up of batch incubation experiments, planktonic anammox biomass was collected from respective MBRs, harvested by centrifugation under anoxic conditions (20°C, ×10000 rpm, 6 min), and washed with anoxic inorganic medium without ammonium and nitrite (purged by N_2_ gas for 1 h) for three times in an anaerobic chamber (Coy Laboratory Products, Michigan, USA). To further purify the MBR anammox cultures, a density gradient separation was performed using Percoll (Cytiva, Tokyo, Japan) as described previously [24]. The Percoll-purified biomass was then anoxically washed twice with inorganic nutrient medium (purged by N_2_ gas for 1 h) without NH_4_^+^ and NO_2_^−^ in the anaerobic chamber, in which anammox bacteria accounted for more than 98% of the total biomass based on FISH analysis. The Percoll-purified biomass was anoxically kept in a 37 °C incubator or room temperature (ca. 25 °C) for overnight before oxygen inhibition or reduction experiments. Percoll purification has been confirmed to have no effect on anammox activity.

### Oxygen inhibition experiments

To determine the inhibitory effect of DO concentrations (µmol L^−1^) on specific anammox activity (SAA), ^15^N-labelling batch incubation experiments were performed at varying O_2_ concentrations (%, v/v) in 20 mL headspace of 70 mL serum vials containing 50 mL of the inorganic medium. The experimental procedure of ^15^N-labelling batch incubation experiments has been described in detail elsewhere [26]. Briefly, the inorganic medium containing 3 mM (^14^NH_4_)_2_SO_4_ and 3 mM Na^15^NO_2_ was firstly purged with N_2_ gas for at least 30 min. Each vial was then sealed with a butyl-rubber stopper and aluminum cap and then autoclaved one night before the start-up of batch incubation. The headspace gas of vials was exchanged by applying 3 cycles of vacuuming (2 min) and purging (1 min at 1.5 atm) with highly pure helium gas (99.9999% He) by a gas exchange machine (Model IP-8, SANSHIN, Yokohama, Japan) to remove the residual air (oxygen) and to enhance the detection sensitivity of ^14+15^N_2_ produced by anammox bacteria. The initial gas pressure of the headspace was set at ca. 1.5 atm. A magnetic stir bar was placed inside the vial to gently stir the medium during batch incubation.

To determine the effect of O_2_ concentrations on SAA, varying volumes of ambient air were injected into the headspace of sealed vials using a gas tight glass syringe (VICI, Baton Rouge, LA, USA) at least 4 h before the inoculation of biomass. The headspace O_2_ concentrations varied from 0 to 3.5% O_2_ (v/v), corresponding to theoretical saturated DO concentrations in the culture medium ranging from 0 to ca. 65 µM and 0 to ca. 53 µM at 25 °C and 37 °C (at 1.5 atm), respectively. To convert the headspace O_2_ concentrations to DO concentrations in the medium, the standard curves of the measured DO concentrations vs. the headspace O_2_ concentrations (%, v/v) were constructed in advance (Fig. S1). The DO concentrations in the medium (µM) were measured by a microsensor (Unisense oxygen needle sensor OX-N 13621) and the Winkler method and plotted against the headspace O_2_ concentrations (%) (Fig. S1). The measured DO concentrations were well correlated with the theoretical O_2_ concentrations at 1.5 atm for both temperatures (25 and 37 °C). The DO concentrations in the following O_2_ inhibition and reduction experiments were determined using the regression lines for microsensor’s data.

For O_2_ inhibition experiments, 1 mL of Percoll-purified biomass suspension was inoculated into the medium in sealed vials to obtain a final biomass concentration of 0.1–0.15 mg-protein mL^−1^. Immediately after biomass inoculation, the air sample (at 0 h) was collected from the headspace, and ^14+15^N_2_ concentrations were measured by a gas chromatography and mass spectrometry (GC-MS, SHIMADZU). The vials with “*Ca*. B. sinica” and “*Ca*. J. caeni” were incubated at 37 °C in an incubator, and the other anammox species were incubated at room temperature (ca. 25 °C) in dark. The ^14+15^N_2_ concentrations were measured hourly, and the maximum specific anammox activity (MSAA, expressed as µmole ^14+15^N_2_ mg-protein^−1^ h^−1^) were calculated by dividing the slope of linear regression of initial ^14+15^N_2_-production as a function of time by the amount of biomass (expressed as protein) in the vial at the beginning of the assay. The lag-phase was considered in the determination of ^14+15^N_2_-production rates. The ^15^N-labelling batch incubation experiments were performed at varying O_2_ concentrations at least triplicate for each species.

The percentages of the MASS (relative MASS) that were maintained after exposure to varying O_2_ concentrations were calculated with respect to the average MASS of nonexposed samples (anoxic cultures) as follows:

1 $$Relative\;MSAA\;\left( \% \right) = \frac{MASS}{{MSAA_{anoxic}}} \times 100$$

where MSAA and MSAA_anoxic_ is the average of MSAA of O_2_-exposed samples and nonexposed samples (anoxic cultures) (µmole ^14+15^N_2_ mg-protein^−1^ h^−1^), respectively.

### Oxygen inhibition model

An oxygen inhibition model with two parameters (IC_50_: the DO concentration that causes 50% inhibition of MSAA (µM) and DO_max_: the DO concentration above which anammox activity is completely inhibited (µM)) were used to quantitatively evaluate the effect of DO concentration on anammox activity [33].

2 $$MSAA = MSAA_{anoxic}\left( {\frac{1}{{1 + \frac{{DO}}{{IC_{50}}}}}} \right)\left( {1 - \frac{{DO}}{{DO_{max}}}} \right)$$

where DO is dissolved oxygen concentration in culture medium (µmole L^−1^). This model was used because the upper limits of DO have been previously reported for marine environments [10, 11] and engineered systems [6]. Values for IC_50_ and DO_max_ were obtained by curve fitting the data.

### Oxygen reduction experiments

The selected anammox species (“*Ca*. B. sinica”, “*Ca*. K. stuttgartiensis” and “*Ca*. Scalindua sp.”) were transferred into 30 mL of the inorganic medium in 100 mL serum vials containing 70 mL headspace. The residual DO was removed and the headspace was exchanged with highly pure helium gas (99.9999% He) at 1.5 atm as described above. O_2_ gas (99%, 200 µL) was injected into the headspace with a gas tight syringe 3 h before the addition of ^15^NH_4_Cl and Na^14^NO_2_ (final concentration of 3 mM each). Thereafter, the vials were incubated at room temperature (ca. 25 °C). The air samples were taken from the headspace over the course of the incubation time, and ^14+15^N_2_ and O_2_ concentrations were measured by a gas chromatography and mass spectrometry (GC-MS, SHIMADZU). The O_2_ concentrations (%) were converted to DO concentration using the standard curves of the measured DO concentrations vs. the headspace O_2_ concentrations (%, v/v) (Fig. S1) as described above. The O_2_ reduction rates were determined by dividing the slope of linear regression of DO concentration as a function of time by the protein concentration and are expressed as µmole DO per g-protein per hour.

### Recovery after oxygen exposures

Anammox biomass was exposed to varying headspace O_2_ concentrations (0 (anoxic), 0.7, 1.4, 2.8, and 21% (v/v)) for 12 h and 24 h in the absence of NH_4_^+^ and NO_2_^−^ as described above. The 21% headspace O_2_ was achieved by unsealing the vials (exposure to ambient air). Unexposed vials (anoxic) were also prepared as a positive control. The oxygen recovery assays were performed in 12 ml vials with a liquid volume of 6 mL at least triplicate biological samples.

After exposure to oxygen for 12 h and 24 h, the headspace gas was exchanged by applying 3 cycles of vacuuming (2 min) and purging (1 min at 1.5 atm) with highly pure helium gas (99.9999% He) by a gas exchange machine (Model IP-8, SANSHIN, Yokohama, Japan). Thereafter, anoxically prepared (N_2_ gas purged) stock solution of (^14^NH_4_)_2_SO_4_ and Na^15^NO_2_ were supplemented to obtain a final concentration of 3 mM each. ^14+15^N_2_ gas production was monitored up to 8–10 h, which was defined as “immediate recovery”. The vials were also continuously incubated for 24 h under anoxic conditions, and then the same amounts of substrates were supplied again (a final concentration of (^14^NH_4_)_2_SO_4_ and Na^15^NO_2_, 3 mM each). The ^14+15^N_2_ gas production was monitored from 24 h to 32 h as described above, which was defined as “secondary recovery”.

### Comparative genome analysis

Comparative genome analysis was carried out to study the types and distributions of genes encoding anti-oxidative enzymes among the anammox species studied. Presence/absence of the genes were examined by performing a blastp search (threshold *e*-value of blastp search 10^−15^) with 31 anammox bacterial genomes affiliated into 18 bacterial species in the bacterial order *Brocadiales* (Table S1). The 18 bacterial species cover all the bacterial species in the order *Brocadiales* defied in the GTDB database (Release RS95) [34]. The genome sequences include those obtained from the enrichment culture of “*Ca*. B. sinica”, “*Ca*. B. sapporoensis”, “*Ca*. J. caeni”, “*Ca*. K. stuttgartiensis” and “*Ca*. Scalindua sp.” used in the present study [35]. Multiple sequence alignment used the ClustalW 1.83 (Gap opening and extension penalties in a pairwise alignment; 10 and 0.05, respectively) [36], and visualized using ESPrint 3.0 [37]. A phylogenetic tree was calculated in MEGA 11.0.8 using maximum likelihood (Jones-Taylor-Thornton model) and neighbor joining methods (Poisson model) [38].

### Anti-oxidative enzyme activity assays

Planktonic anammox cells were collected from respective stock MBRs and incubated in 70 mL vials after Percoll density gradient centrifugation. During incubation, anammox cells were subjected to two patterns of oxygen exposure: (i) exposure to different O_2_ concentrations (0, 0.7, 1.4, and 2.8% (v/v)) for 12 h; or (ii) exposure to air-saturating DO concentrations (ambient air) for different periods of time (0, 0.5, 1 and 2 h). After each oxygen exposure, biomass was harvested by centrifugation (4 °C, 10,000 rpm, 6 min) and concentrated 25-fold by resuspending in an ice-cold potassium phosphate buffer (50 mM, pH 7.4). To prevent protein degradation, 0.1 mM phenylmethylsulfonyl fluorid (PMSF) was added as a protease inhibitor. The cell suspension was disrupted by passing thrice through a French pressure cell press unit (AVESTIN, ON, Canada) at homogenizing pressure of 1200 MPa. Cell debris was removed by centrifugation (4 °C, 4500 rpm, 60 min). The resulting supernatant fraction was transferred to 15 mL sterile tubes and stored in an ice-cold bath. All cell-free extracts were tested within a day. At least, three independent determinations were performed for each enzyme activity in all conditions. All anti-oxidative enzyme activities were expressed as units of enzyme activity per milligram of protein of the cell-free extract.

Superoxide dismutase (Sod) activity was assayed spectrophotometrically at 560 nm by measuring its ability to inhibit the photochemical reduction of nitroblue tetrazolium (NBT) (superoxide anion O_2_^•-^ reacts with NBT and reduces the yellow tetrazolium to a blue precipitate) according to the protocol previously described [39]. Since the assays were conducted in microplates, the reaction mixture contained in 200 µL: 50 mM potassium phosphate buffer (pH 7.8), 10 mM _L_-methionine, 60 µM NBT, 8 µM EDTA, 1.6 µM riboflavin, and 22 µL of cell-free extract (0.05–0.06 mg-protein). Riboflavin was added at the end, and the tubes were mixed by shaking [40]. The microplate was illuminated by a 4000 Lux cool LED lightboard (AXEL, Tokyo, Japan) for 20 min, and the absorbance at 560 nm was recorded at 2, 4, 6, 8, 10, 15, and 20 min in a multilabel plate counter (PerkinElmer, Waltham, USA). The samples containing the same components were placed in dark, which were used as a blank reference. One activity unit (1 U) of Sod was defined as the amount of enzyme required to inhibit the rate of riboflavin and illumination dependent NBT reduction by 50% at 25 °C, pH 7.8, and a light intensity of 4000 Lux [40].

Catalase (Cat) activity was assayed spectrophotometrically at 240 nm by measuring its ability to decompose H_2_O_2_ as described elsewhere [41]. The reaction mixture contained in 1 mL: 50 mM potassium phosphate buffer (pH 7.0), 10 mM H_2_O_2_, and 0.1 mL of cell-free extracts (0.2–0.3 mg-protein). The reaction was initiated by adding H_2_O_2_ into a quartz cuvette containing phosphate buffer and enzyme extracts. One activity unit (1 U) of Cat was defied as the amount of enzyme required to catalase the decomposition of 1 µmol H_2_O_2_ in 1 min at 25 °C and pH 7.0.

Cytochrome *c* peroxidase (Ccp) activity was assayed spectrophotometrically at 550 nm by monitoring the loss of reduced cytochrome *c* according to the protocol provided by Munkres et al. [42]. The reaction mixture contained in 1 mL: 50 mM acetate buffer (pH 6.0), 1 mM EDTA, 1 mM sodium azide, 20 µL of a reduced cytochrome c solution (1.2 mg mL^−1^), 180 µM H_2_O_2_, and 0.1 mL of cell-free extracts (0.2–0.3 mg-protein). The net rate of absorbance change was obtained by subtracting the rate of a control without cell-free extract. One activity unit (1 U) of Ccp was defended as the amount of enzyme required to catalase the oxidation of 1 µmol cytochrome *c* in 1 min at 25 °C and pH 6.0.

Glutathione peroxidase (Gpx) activity was assayed spectrophotometrically at 340 nm by monitoring the loss of NADPH according to the protocol provided by Drotar et al., [43]. The reaction mixture contained in 1 mL: 50 mM potassium phosphate buffer (pH 7.0), 2 mM EDTA, 2 mM reduced glutathione, 0.1 mM NADPH, 2.5 units of glutathione reductase, 0.09 mM H_2_O_2_, and 0.1 mL of cell-free extracts (0.2–0.3 mg-protein). The net rate of absorbance change was obtained by subtracting the rate of a control without cell-free extract. One activity unit (1 U) of Gpx was defined as the amount of enzyme required to catalase the reduction of 1 µmol NADPH in 1 min at 25 °C and pH 7.0.

### Chemical analyses

For determination of ^14+15^N_2_ gas and O_2_ concentrations, 50 μL of headspace gas was collected from the tested vial with a gas tight syringe (VICI, Baton Rouge, LA, USA) and injected into a gas chromatography mass spectroscopy (GCMS-QP2010SE, Shimadzu, Japan) equipped with a CP-Pora Bond Q fused silica capillary column (Agilent Technologies, Santa Clara, CA, USA) as described previously [25]. The specific anammox activity (SAA) was calculated by dividing the ^14+15^N_2_ gas production rate by biomass (protein) concentration. In each experiment, SAA tests were performed in triplicate at least.

Concentrations of DO were measured by a Unisense oxygen needle sensor (OX-N 13621, Aarhus, Denmark). The oxygen sensor was calibrated using a two-point calibration (zero and a saturation point). The deionized water was purged with pure N_2_ gas for at least 1 h or dissolving 1 g of sodium sulfite (Na_2_SO_3_) to obtain a zero-DO solution and bubbled with air for at least 1 h to achieve the saturated oxygen concentration at 25 °C and 37 °C, respectively.

Biomass concentration was determined as protein concentration with the DC Protein Assay Kit (Bio-Rad Laboratories, Munich, Germany) using the bovine serum albumin (BSA) as the protein standard. In brief, 1 mL of cell suspension was collected, centrifuged at 12,000 rpm for 15 min, resuspended in the same amount of 10% (w/v) sodium dodecyl sulfate (SDS) solution and incubated for 30 min at 99 °C (Note this procedure is unnecessary for the protein measurement of cell-free extract in enzyme activity tests). Then, 5 µL of the cell suspension was incubated with Bio-Rad protein assay reagent in microplate for 15 min at 25 °C. The absorbance was measured at 750 nm.

## Results and Discussion

### Effect of O_2_ concentration on specific anammox activity (SAA)

**Fig. 1:**
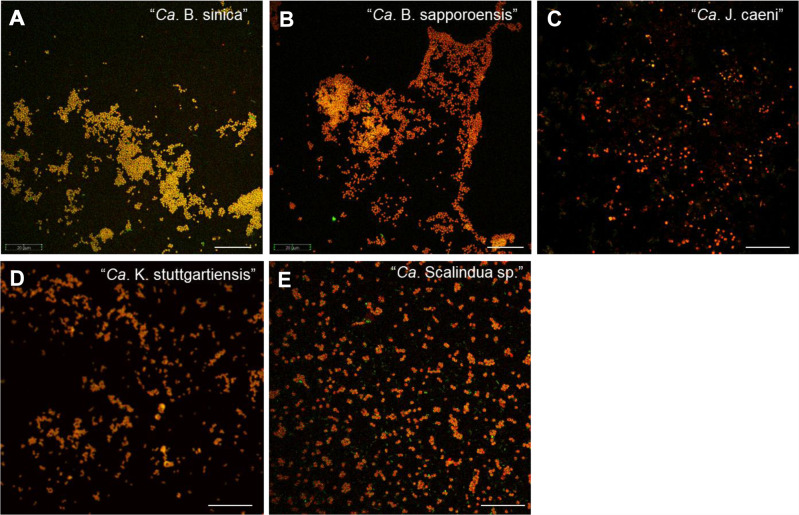
FISH images of Percoll-purified planktonic anammox bacterial cells. Cells were hybridized with combination of FITC-labeled EUB338 mix probe (most of the *Eubacteria*) and an anammox species specific probe; Alexa555-labeled AMX156 probe for “*Ca*. B. sinica” (**A**) and “*Ca*. B. sapporoensis” (**B**), TRITC-labeled JEC152 probe for “*Ca*. J. caeni” (**C**), CY3-labeled KST157 probe for “*Ca*. K. stuttgartiensis” (**D**), and TRITC-labeled Scal1129b probe for “*Ca*. Scalindua sp.” (**E**), respectively. Anammox bacteria were shown in orange yellow (green+red), whereas other bacteria were shown in green. All FISH images show highly enriched and well dispersed cells. Scale bars represent 20 µm.

It was confirmed that the Percoll-purified anammox biomass were highly enriched and well dispersed (Fig. 1). The high purity (> 98%) was also confirmed for all anammox species by FISH and the measurements of 16 S rRNA gene copy numbers by qPCR.

**Fig. 2:**
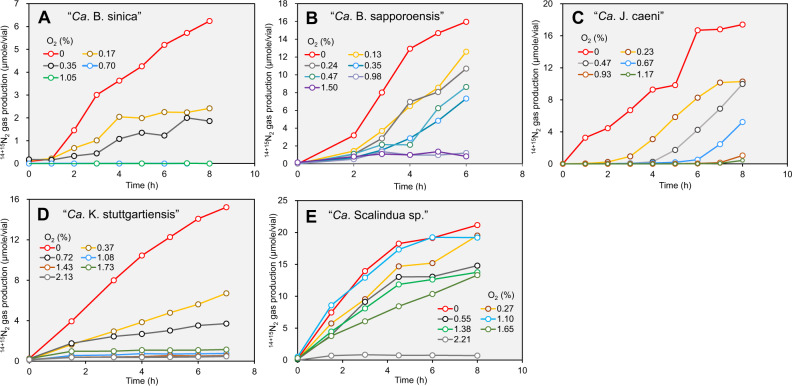
Typical time courses of ^14+15^N_2_ productions determined at different O_2_ concentrations (v/v, %) for five different anammox species. “*Ca*. B. sinica” (**A**), “*Ca*. B. sapporoensis” (**B**), “*Ca*. J. caeni” (**C**), “*Ca*. K. stuttgartiensis” (**D**), and “*Ca*. Scalindua sp.” (**E**). Batch incubation experiments with ^15^NO_2_^−^ (3 mM) and ^14^NH_4_^+^ (3 mM) were performed at varying headspace O_2_ concentrations for each species at least triplicate. One of representative data sets is presented here.

The Percoll-purified planktonic anammox cells were cultured with ^14^NH_4_^+^ and ^15^NO_2_^−^ in sealed vials with headspace containing different O_2_ concentrations (up to 3.32%, corresponding to ca. 60 µM and 50 µM dissolved O_2_ (DO) at 25 °C and 37°C at 1.5 atm, respectively), and then ^14+15^N_2_ production rates were measured for five anammox species (Fig. 2). DO concentrations in the vials gradually decreased during the batch incubations for all batch experiments (Fig. S2). The onset of ^14+15^N_2_ gas production was gradually delayed (lag-time), and the ^14+15^N_2_ gas production rates decreased along with the increase of O_2_ concentration for 4 freshwater anammox species (Fig. 2). However, a marine species “*Ca*. Scalindua sp.” could steady produce ^14+15^N_2_ up to 1.65% O_2_ (DO = ca. 30 µM) without any lag time.

**Fig. 3:**
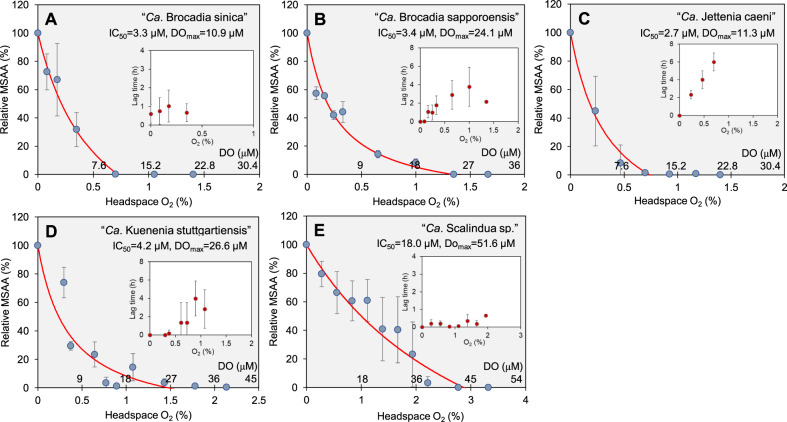
Effect of O_2_ concentrations on relative maximum specific anammox activity (MSAA) for five different anammox species. “*Ca*. B. sinica” (**A**), “*Ca*. B. sapporoensis” (**B**), “*Ca*. J. caeni” (**C**), “*Ca*. K. stuttgartiensis” (**D**), and “*Ca*. Scalindua sp.” (**E**). The MSAA (µmole ^14+15^N_2_ mg-protein^−1^ h^−1^) was calculated from maximum ^14+15^N_2_ production rate and amount of biomass in the vial and given as a percentage of MSAA observed at DO = 0 µM (defined as 100%). The red lines represent DO inhibition kinetics as shown in Eq. (2) fitted to the data. Inserted figures show the effect of DO concentrations on lag times of ^14+15^N_2_ gas production after exposures to O_2_. The lag time means the time needed to reduce DO concentration to tolerable levels and to repair and reactivate damaged enzyme systems. DO concentrations were calculated based on the standard curves of the measured DO concentration (µM) vs. the headspace O_2_ concentration (%) at 25 °C and 37 °C at 1.5 atm, respectively (Fig. S1, see Materials and Methods). Error bars represent the standard deviations of three replicate samples.

The effect of O_2_ concentration on the MSAA, as indicated by relative MSAA (%), was evaluated (Fig. 3). The relative MSAA dramatically decreased with increasing O_2_ concentrations, approaching zero asymptotically, for freshwater anammox species. No significant anammox activity was virtually detected at >1.0% O_2_ (corresponding to ca.15.2 µM and 18 µM DO at 25 °C and 37 °C, respectively) for freshwater anammox species. In contrast, the MSAA of “*Ca*. Scalindua sp.” could be detected up to 2.0% O_2_ (36 µM DO), showing the least O_2_ sensitivity among the anammox species examined in the present study.

The DO concentrations that cause 50% inhibition of MSAA (IC_50_) and the maximum DO concentrations above which anammox is completely inhibited (DO_max_) were estimated for each anammox species based on the inhibition model Eq. (2). All freshwater anammox species showed similar responses to increased O_2_ (high sensitivity). The IC_50_ values were in the range 2.7 - 4.2 µM, and DO_max_ values were in the range 10.9 - 26.6 µM. In contrast, a marine anammox species, “*Ca*. Scalindua sp.” exhibited much higher values of IC_50_ = 18.0 µM and DO_max_ = 51.6 µM without significant lag times. The MSAA of “*Ca*. Scalindua sp.” was inhibited by only ca. 30% at 10 µM DO (the DO upper limit of suboxic condition [44]).

A wide range of oxygen tolerance capabilities have been reported for freshwater anammox species so far [6]. For example, DO_max_ was reported to be ~200 µM for “*Ca*. K. stuttgartiensis”, < 63 µM for “*Ca*. B. sinica”, < 1 µM for “*Ca*. B. anammoxidans”, 120 µM for “*Ca*. B. caroliniensis”, and 70 µM for “*Ca*. B. fulgida”, respectively. On the other hand, complete inhibition was observed at much lower levels of DO (1.25–3.75 µM) in lab-scale bioreactors [6, 12–14]. The reason for the variation in reported values could be partly attributed to formation of microbial aggregates and the presence of coexisting aerobes (i.e., the purity of anammox biomass). Therefore, intrinsic oxygen tolerance capabilities of these anammox bacteria cannot be simply evaluated and compared with these reported data.

In the present study, it was confirmed that since highly enriched (>98%) and well-dispersed cells were subjected to the oxygen inhibition studies, the influence of oxygen consumption by coexisting aerobes and/or oxygen shielding effects of aerobes in microbial aggregates can be excluded. Therefore, the observed oxygen tolerances could conceivably be their intrinsic properties. Anammox activities of freshwater species (“*Ca*. B. sinica”, “*Ca*. B. sapporoensis”, “*Ca*. J. caeni”, and “*Ca*. K. stuttgartiensis”) were completely inhibited at ~25 µM DO with IC_50_ of 2.7–4.2 µM DO, which are in the middle of the reported range.

The previous O_2_ amendment studies have shown that anammox activities of oceanic water samples (i.e., Namibian OMZ, Black sea, and Peruvian OMZ) were 50% inhibited at ~ 0.9 µM [4], ~ 8 µM [10], and 1.9 to 16 µM DO [11], respectively. The upper DO limits for anammox in marine systems were reported to be ~20 µM [9, 11, 45] or slightly lower ~10 µM [4, 8, 10]. The IC_50_ and DO_max_ values of “*Ca*. Scalindua sp.” obtained in the present study were higher than these previously reported values. One of the reasons could be due to that much higher cell density (~10^9^ - 10^11^ copies mL^−1^) were utilized in the present study than those O_2_ amendment studies of oceanic OMZ samples (usually ~10^4^–10^6^ copies mL^−1^) [9, 45]. Since anammox activity is highly dependent on cell density [46, 47], lower cell density makes them more susceptible to oxygen inhibition (Fig. S3). Similar inoculum size-dependent oxygen tolerance ability was previously reported for an obligatory anaerobe, *Clostridium butyricum* [48].

Another reason could be that since planktonic free-living “*Ca*. Scalindua sp.” has been continuously cultured for more than 10 years in MBRs in our laboratory, in which it is difficult to maintain strict anoxic conditions, oxygen insensitive *Scalindua* cells could have been selectively enriched during such a long cultivation period. Since anammox bacterial cells in our MBRs are highly enriched and planktonic biomass, not aggregated one, as shown in FISH images (Fig. 1), oxygen shielding effect and/or oxygen consumption by coexisting planktonic aerobes seems to be minimum.

The experimental results clearly suggest that a marine anammox species “*Ca*. Scalindua sp.” intrinsically possess higher oxygen tolerance than freshwater species. More importantly, the upper DO limit for “*Ca*. Scalindua sp.” was much higher than the values reported so far (~20 µM). This might suggest that the ocean volume, where N_2_ production (N loss) by anammox is potentially expected, could be larger than we have been led to believe so far.

### Oxygen reduction rates

**Fig. 4:**
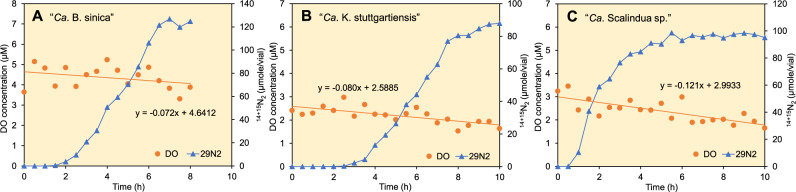
Determination of oxygen reduction rates. The selected “*Ca*. B. sinica” (**A**), “*Ca*. K. stuttgartiensis” (**B**), and “*Ca*. Scalindua sp.” (**C**) were incubated with ^15^NO_2_^−^ (3 mM) and ^14^NH_4_^+^ (3 mM) in the presence of oxygen at room temperature (ca. 25 °C). The headspace ^14+15^N_2_ and O_2_ concentrations were measured with GC/MS. DO concentrations were calculated based on the standard curves of the measured DO concentration (µM) vs. the headspace O_2_ concentration (%) at 25 °C and 1.5 atm (Fig. S1, see Materials and Methods). The protein concentration was 0.28, 0.15, and 0.47 mg-protein/mL for “*Ca*. B. sinica”, “*Ca*. K. stuttgartiensis”, and “*Ca*. Scalindua sp.”, respectively. The batch experiments were conducted in duplicates. One of representative data sets is presented here.

“*Ca*. B. sinica”, “*Ca*. K. stuttgartiensis”, and “*Ca*. Scalindua sp.” were cultured in the presence of oxygen (ca. 0.18–0.20% headspace O_2_). For all three anammox species, O_2_ gradually decreased with the incubation time while producing ^14+15^N_2_ in batch cultures (Fig. 4). The O_2_ reduction rates were determined by dividing the slope of linear regression of DO concentration by the protein concentration. The oxygen reduction rates of “*Ca*. B. sinica” and “*Ca*. Scalindua sp.” were similar (0.26 nmole O_2_/g-protein/h), whereas “*Ca*. K. stuttgartiensis” reduced O_2_ at a more rapid rate (0.53 nmole O_2_/g-protein/h). The specific ^14+15^N_2_ production rates of “*Ca*. B. sinica”, “*Ca*. K. stuttgartiensis”, and “*Ca*. Scalindua sp.” were 3.31 µmole N_2_/g-protein/h, 3.91 µmole N_2_/g-protein/h, and 3.44 µmole N_2_/g-protein/h, respectively. The O_2_ reduction rates were 4 orders of magnitude lower than the specific ^14+15^N_2_ production rate, suggesting that O_2_ could be reduced for detoxification not for respiration. As discussed below (Section of comparative genome analysis), O_2_ can be enzymatically reduced to H_2_O by nonrespiratory proteins for detoxification.

### Recovery of anammox activity after O_2_ exposure

**Fig. 5:**
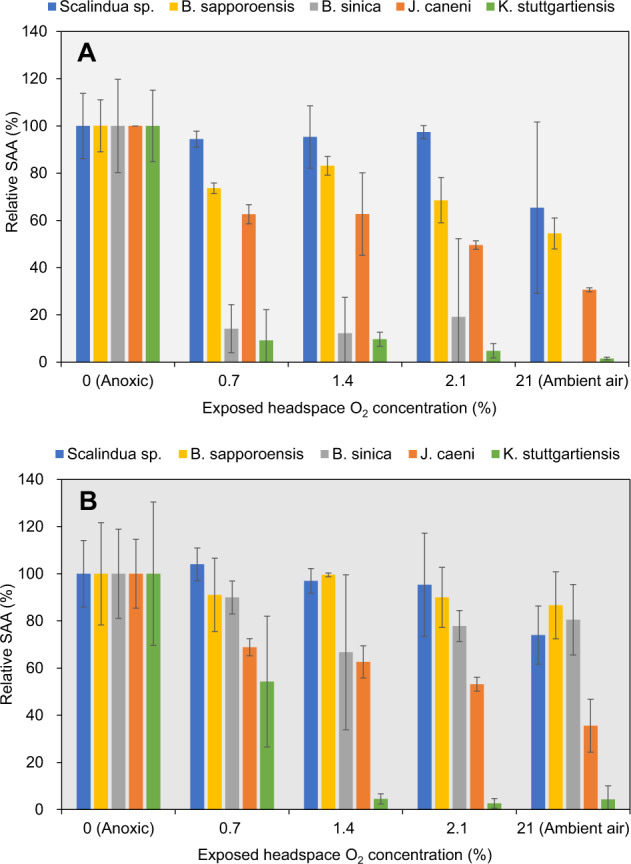
Recovery of MSAA after exposure to different O_2_ concentrations for 12 h. After 12 h exposure to different headspace O_2_ concentrations, relative maximum specific anammox activities (MSAA) were determined immediately (**A**) and after 24-h anoxic incubation (**B**) in the absence of NH_4_^+^ and NO_2_^−^. The initial MSAA measured under anoxic condition before O_2_ exposure was defined as 100% (Anoxic). The data represent the average ± standard deviations of triplicate samples. It should be noted that “*Ca*. B. sapporoensis” formed small flocs in this recovery experiments, and that the biomass concentrations of “*Ca*. J. caeni” were about 10 times higher than other species. These could be reasons for higher recovery from O_2_ exposure.

After 12-h exposure to varying headspace O_2_ concentrations (0, 0.7, 1.4, 2.1, and 21% (ambient air) O_2_ at 1.5 atm) in the absence of NH_4_^+^ and NO_2_^−^, the headspace gas was exchanged with highly pure Helium gas (> 99.9999%) by vacuuming and purging 3 times to restore anoxic conditions. (^14^NH_4_)_2_SO_4_ (3 mM) and Na^15^NO_2_ (3 mM) were supplemented at 0 h, and the recovery of SAA (^14+15^N_2_ production rate) was examined (Fig. S4). The ^14+15^N_2_ production was immediately detected without any lag time for “*Ca*. Scalindua sp.” even after exposure to ambient air (21% O_2_) for 12 h. Subsequent anoxic incubation resulted in almost full recovery: 94 ± 3%, 95 ± 13%, 97 ± 3%, and 65 ± 36% in MSAA after exposure to 0.7%, 1.4%, 2.1 %, and 21% (ambient air) O_2_ for 12 h, respectively (Fig. 5A). In contrast, “*Ca*. B. sinica” and “*Ca*. K. stuttgartiensis” were severely inhibited by all O_2_ concentrations and could not recover within 7 h anoxic incubation. However, “*Ca*. B. sinica” exposed to 21% O_2_ for 12 h regained 80 ± 15% of the initial activity 13 h after the restoration of anoxic incubation, whereas “*Ca*. K. stuttgartiensis” could not recover at all, indicating irreversible inhibition (Fig. S5). “*Ca*. J. caeni” gradually recovered 42 ± 2% of initial activity after exposed to 21% O_2_ with lag times (ca. 0.5–1.5 h). It should be noted that the *Jettenia* biomass concentrations were about 10 times higher than other species, which could result in the quicker and better recovery. “*Ca*. B. sapporoensis” exhibited an immediate recovery of ^14+15^N_2_ production, however, this could be due to the formation of small flocs during the recovery test, in which O_2_ transfer limitation could shield anammox bacteria from O_2_ exposure and thus anammox bacteria could remain active inside.

After 24-h anoxic incubation, the SAAs further recovered > 40% of anoxic control activity, except for “*Ca*. K. stuttgartiensis” (Fig. 5B). This high oxygen tolerance is partly because the oxygen sensitivity and reversibility of the oxygen inhibition were assessed in the absence of NH_4_^+^ and NO_2_^−^ in the present study, indicating that cells were metabolically inactive. The metabolically inactive cells are likely less susceptible to oxygen inhibition than active cells because of the minimum production of NAD(P)H, which is probably acting as electron donor for O_2_ reduction (*i.e*., production of reactive oxygen species, ROS) [49].

The effect of longer O_2_ exposure time (24 h) on the MSAA recovery was also examined in the absence of NH_4_^+^ and NO_2_^−^ under ambient air (Fig. S6). The ^14+15^N_2_ profiles after 24 h-exposure to ambient air exhibited a recovery trend similar to 12 h-exposure (Fig. S5). “*Ca*. Scalindua sp.” immediately reinitiated almost the same activities as anoxic controls even after 24 h exposure to ambient air. Interestingly, “*Ca*. B. sinica” gradually increased to 80 ± 15% of the initial activity after 18 h lag time, whereas “*Ca*. K. stuttgartiensis” could not recover during > 30 h of anoxic incubation. These results coincided with those of the 12 h-exposure test (Fig. S5).

These experimental results suggest that anammox bacteria were aerotolerant anaerobes, although anammox bacteria are previously classified as strict anaerobes. A marine species, “*Ca*. Scalindua sp.”, exhibited the highest aerotolerance and reversibility among anammox species studied. Furthermore, the oxygen inhibition was reversible except for “*Ca*. K. stuttgartiensis”. The reversibility seems to depend on the exposure conditions; reversible at low DO levels (0.25–2% O_2_) but probably irreversibly inhibited at high DO levels (> 46.9 µM or 20% O_2_) for freshwater anammox species [12–14]. The O_2_ toxicity arises directly from itself or from reactive oxygen species (ROS). O_2_ and/or ROS reacts unspecifically with catalytic centres or accessory metals of redox enzymes and proteins. For example, key enzymes involved in energy metabolism in anaerobes such as pyruvate ferrodoxin oxidoreductase (PFOR), pyruvate formate lyase (PFL), CO-dehydrogenease, 4-hydroxy-butyryl-CoA dehaydratase, and proteins containing low potential iron-sulfur (Fe-S) clusters are known to be O_2_ sensitive [50, 51]. However, unfortunately transcriptomic and/or proteomic studies have not been conducted to confirm that these enzymes or proteins were indeed inactivated by molecular O_2_ and repaired after the restoration of anoxic incubation in the present study.

In the present study, since the anammox biomass was highly enriched and well dispersed, the obtained responses to O_2_ exposure and recovery from O_2_ inhibition are most likely intrinsic oxygen tolerance capability of anammox species. For granular biomass, co-existing micro-aerobic heterotrophic bacteria and O_2_ transfer limitation could shield anammox bacteria from O_2_ exposure, resulting in higher resilience as compared to planktonic free-living anammox bacteria. For example, floc-style *“Brocadia”* biomass (dominated only 75%) reinitiated activity to 55-80% of pre-exposed activity after exposure to air-saturated DO (ca. 250 µM) for 24 h [14].

### Comparative genome analysis

#### ROS detoxification mechanisms

Upon exposure to O_2_, anaerobes need to detoxify O_2_ and generated ROS (e.g., superoxide anion (O_2_^•-^) and hydrogen peroxide (H_2_O_2_)) for survival [52]. O_2_ can be enzymatically reduced to H_2_O by nonrespiratory flavodiiron proteins (Fdp), named rubredoxin:oxygen oxidoreductase (Roo) [50, 53], reverse rubrerythrins (revRbr) [50, 54], and/or terminal oxidase [55] (Fig. S7A). Fdps (Roo) and revRbr are widely distributed among anaerobic or microaerophilic bacteria and archaea but not in aerobes [50]. Since O_2_^•-^ is more toxic than H_2_O_2_, the detoxification of O_2_^•-^ is a pivotal function in O_2_ tolerance. O_2_^•-^ can be converted to H_2_O_2_ and O_2_ by superoxide dismutase (Sod), which plays a key role in ROS detoxification. The generated H_2_O_2_ can be further converted to H_2_O and O_2_ by catalase (Cat). In general, aerobes and facultative anaerobes utilize the canonical Sod-Cat detoxification system with O_2_ generation.

Alternatively, O_2_^•-^ can be reduced to H_2_O_2_ by superoxide reductase (Sor) such as neelaredoxin (Nlr) and desulfoferrodoxin (Dfx) with reduced rubredoxin (Rd_red_) as an electron donor [56] (Fig. S7B). The oxidized rubredoxin (Rd_ox_) is reduced back to the reduced form by NADH-dependent rubredoxin oxidoreductase (Nror). The generated H_2_O_2_ is reduced to H_2_O by peroxidase such as cytochrome *c* peroxidases (Ccps) and rubrerythrins (Rbrs).

$${{{{{{{\mathrm{O}}}}}}}}_2^{ \bullet - } + 2{{{{{{{\mathrm{H}}}}}}}}^ + + {{{{{{{\mathrm{Rd}}}}}}}}_{{{{{{{{\mathrm{red}}}}}}}}} \to {{{{{{{\mathrm{H}}}}}}}}_2{{{{{{{\mathrm{O}}}}}}}}_2 + {{{{{{{\mathrm{Rd}}}}}}}}_{{{{{{{{\mathrm{ox}}}}}}}}}\left( {{{{{{{{\mathrm{mediated}}}}}}}}\;{{{{{{{\mathrm{by}}}}}}}}\;{{{{{{{\mathrm{Sor}}}}}}}}} \right)$$

$${{{{{{{\mathrm{Rd}}}}}}}}_{{{{{{{{\mathrm{ox}}}}}}}}} + 0.5\;{{{{{{{\mathrm{NADH}}}}}}}} \to 0.5\;{{{{{{{\mathrm{NAD}}}}}}}}^ + + {{{{{{{\mathrm{Rd}}}}}}}}_{{{{{{{{\mathrm{red}}}}}}}}}\left( {{{{{{{{\mathrm{mediated}}}}}}}}\;{{{{{{{\mathrm{by}}}}}}}}\;{{{{{{{\mathrm{Nror}}}}}}}}} \right)$$

$${{{{{{{\mathrm{H}}}}}}}}_2{{{{{{{\mathrm{O}}}}}}}}_2 + {{{{{{{\mathrm{RH}}}}}}}}_2 \to 2{{{{{{{\mathrm{H}}}}}}}}_2{{{{{{{\mathrm{O}}}}}}}} + {{{{{{{\mathrm{R}}}}}}}}\;\left( {{{{{{{{\mathrm{mediated}}}}}}}}\;{{{{{{{\mathrm{by}}}}}}}}\;{{{{{{{\mathrm{peroxidases}}}}}}}}} \right)$$

The electron donor of Ccps and Rbrs is cytochrome *c* and Rd_red_, respectively. The Sor-Ccp and/or Rbr detoxification system confers a selective advantage on anammox bacteria because O_2_ is not generated.

All anammox species possess the genes encoding the class A Fdp [57] with the N-terminal methallo-*β*-lactamase domain and the C-terminal flavodoxin domain and rubrerythrin, which were considered to function in O_2_ and/or NO detoxification [58, 59] (Table 1, Table S1). Intriguingly, only the *Scalindua* genomes (except for the *Scalindua* sp001828595) contained an operon encoding Fdp and rubredoxin (Rd) which is a potential electron carrier of Fdp. In the operon, the *rd* gene is located downstream of the *fdp* gene, suggesting the expression of *Scalindua* *fdp* and *rd* is regulated under the same regulation factor. As for the terminal oxidase, the *Scalindua* and *Kuenenia* genomes have the genes encoding *cbb*_*3*_-type cytochrome *c* which can be involved in O_2_ reduction [60]. Cytochrome *ba3* oxidase and cytochrome oxidase were not identified in all anammox species. In addition, the gene encoding a flavorubredoxin (flavoRd) with a NO-binding non-heme diiron center was identified in all anammox genomes, which possibly function in NO and/or O_2_ reduction and detoxification [61, 62]. The gene encoding Nror with N-terminal FAD/NAD-binding domain and C-terminal Rd-binding domain [63, 64] was identified in the *Scalindua*, *Jettenia*, and *Brocadia* genomes.

**Table 1:** Genes encoding known anti-oxidative enzymes in anammox species studied in the present study.

blastp query		*Scalindua* sp.	*K. stuttgartiensis*	*J. caeni*	*B. sinica*	*B. sapporoensis*
Superoxide dismutase (Sod)						
Mn-type	*E. coli* (*sodA*, b3908)	SCALA7_17830				
Fe-type	*E. coli* (*sodB*, b1656)	SCALA7_17830				
putative Sod	*K. stuttgartiensis* (kustd1303)		HKUEST01_01390	JETCAE04_31330 JETCAE04_04310	+	HBSAPP01_09280
Cu/Zn-type	*E. coli* (*sodC*, b1646)					
Ni-type	*S. seoulensis* (NiSOD, D0Z67_19685)					
Superoxide reductase (Sor)						
Neelaredoxin (Nlr)	*P. furiosus* (*nlr*, PF1281)					
	*A. fulgidus* (*nlr*, AF_0344)					
	*K. stuttgartiensis* (kustc0565)	SCALA7_12610	HKUEST01_26280		+	HBSAPP01_09840
Desulfoferrodoxin (Dfx)	*D. vulgaris* (*dfx*, DVU_3183)					
Rubredoxin:oxygen oxidoreductase (Roo)						
Flavodiiron proteins (Fdp)	*D. vulgaris* (*fdp*, DVU_3185)	SCALA7_01450 SCALA7_19040 SCALA7_23040 SCALA7_11410	HKUEST01_04980 HKUEST01_29320	JETCAE04_28610 JETCAE04_13290	HBSIN01_06970 HBSIN01_03670	HBSAPP01_18710 HBSAPP01_19560
	*C. difficile* 630 (*fdp1*, cd1157)	SCALA7_19040 SCALA7_11410 SCALA7_23040 SCALA7_01450	HKUEST01_29320 HKUEST01_04980	JETCAE04_13290 JETCAE04_28610	HBSIN01_03670 HBSIN01_06970	HBSAPP01_19560 HBSAPP01_18710
	*C. difficile* 630 (*fdp2*, cd1623)	SCALA7_11410 SCALA7_19040 SCALA7_23040 SCALA7_01450	HKUEST01_29320 HKUEST01_04980	JETCAE04_13290 JETCAE04_28610	HBSIN01_03670 HBSIN01_06970	HBSAPP01_19560 HBSAPP01_18710
Fdp-rubredoxin (Rd) operon	SCALA7_01450 SCALA7_01460					
Rubrerythrins (Rbr)	*D. vulgaris* (*rbr1*, DVU_3094)	SCALA7_05050	HKUEST01_31710 HKUEST01_10970	JETCAE04_03240 JETCAE04_29250	HBSIN01_02670 HBSIN01_01200	HBSAPP01_08180
	*D. vulgaris* (*rbr2*, DVU_2310)					
	*C. difficil* 630 (*rbr1*, cd1474)	SCALA7_36590				
	*C. difficil* 630 (*rbr2*, cd1524)	SCALA7_36590				
Flavorubredoxin (flavoRd)						
	*E. coli* nitric oxide reductase flavorubredoxin (*norV*, b2710)	SCALA7_01450 SCALA7_19040 SCALA7_11410 SCALA7_23040	HKUEST01_04980 HKUEST01_29320	JETCAE04_28610 JETCAE04_13290	HBSIN01_06970 HBSIN01_03670	HBSAPP01_18710 HBSAPP01_19560
NADH:rubredoxin oxidoreductase (Nror)^*^						
	*C. acetobutylicum* NADH:rubredoxin oxidoreductase (CA_C2448)	SCALA7_22340		JETCAE04_31200	HBSIN01_17260	HBSAPP01_09890
	*E. coli* NADH:flavorubredoxin reductase (*norW*, b2711)			JETCAE04_31200	HBSIN01_17260	HBSAPP01_09890
Catalase (Cat)						
	*E. coli* (*katE*, b1732)	SCALA7_02240	HKUEST01_01410		HBSIN01_19720	
Perooxidase						
	*E. coli* cytochrome *c* perooxidase (*ccp*, b3518)	SCALA7_32150	HKUEST01_29510 HKUEST01_00680	JETCAE04_31260	HBSIN01_23990 HBSIN01_24990	HBSAPP01_09230 HBSAPP01_09910
	*E. coli* glutathione perooxidase (*btuE*, b1710)	SCALA7_22180 SCALA7_18270				
Terminal oxidase						
	*K. stuttgartiensis cbb*_*3*_-type cytochrome *c* oxidase subunit (*ccoP*, kustc0427)	SCALA7_32500	HKUEST01_21110			
	*K. stuttgartiensis* unknown protein (kustc0428)	SCALA7_32510	HKUEST01_21120			
	*K. stuttgartiensis cbb*_*3*_-type cytochrome *c* oxidase subunit 1 (*ccoN*, kustc0429)	SCALA7_32520	HKUEST01_21130			
	K. stuttgartiensis *cbb*_*3*_-type cytochrome *c* oxidase maturation protein (*ccoS*, kustc0430)					
	*E. coli* cytochrome *bd*-I ubiquinol oxidase subunit X (*cydX*, b4515)					
	*E. coli* cytochrome *bd* ubiquinol oxidase subunit I (*cydA*, b0733)					
	*E. coli* cytochrome *bd* ubiquinol oxidase subunit II (*cydB*, b0734)					
	*Thermus thermophilus ba*3-type cytochrome *c* oxidase polypeptide I (TTHA1135)					
	*Thermus thermophilus ba*3-type cytochrome *c* oxidase polypeptide II (TTHA1134)					

"+"; not found in the genome sequence but found in other genome sequence affiliated into the same bacterial species. *The protein with N-terminal FAD/NAD-binding domain and rubredoxin-binding C-terminal domain.

The gene encoding putative Sod (the kustd1303 protein) was widely conserved in the genomes of *B. sinica*, *B. sapporoensis*, *J. caeni*, and *K. stuttgartiensis*, and also in the other *Brocadiaceae* genomes except for those affiliated into the *Brocadiaceae*-family (Table S1). However, only the *Scalindua* sp. (affiliated into the species SCALAELEC01 sp004282745) and other *Scalindua* genomes (*Scalindua* sp001828595 and *Scalindua japonica*) have a gene encoding typical Fe/Mn-type Sod (*sodA* or *sodB*) (Table 1). Multiple sequence alignment of the anammox bacterial Sod showed that all the metal binding sites were conserved among the *Scalindua* Sod (Fig. S8). On the other hand, all the *Brocadiaceae* Sod lacked the histidine residues requiring for the metal binding (*i.e*., His_24_ and His_75_), and N-terminal alpha-hairpin domain was also not conserved in the *Brocadiaceae* Sod. Furthermore, phylogenetic analysis of anammox bacterial Sod revealed that *Scalindua* Sod and *Brocadiaceae* Sod are affiliated into different phylogenetic clades (Fig. S9). These evidences indicate the *Brocadiaceae* Sod is likely not to function.

As for O_2_^•-^ reduction to H_2_O_2_ by Sor, the gene encoding neelaredoxin (Nlr) with functionally-important amino acid residues [65] (Fig. S10) was widely conserved among the anammox bacterial genomes except for *Jettenia* (Table S1). As for reduction of the generated H_2_O_2_, all genera possess the genes encoding cytochrome *c* peroxidase (Ccp) that obtains reducing equivalents from cytochrome *c*, whereas *B. sinica, K. stuttgartiensis*, and *Scalindua* sp. possess the genes encoding catalase (Cat). The *B. sinica* and *Scalindua* sp. Cat were affiliated into a phylogenetic clade apart from that of *K. stuttgartiensis* Cat (Fig. S11). The genes encoding another peroxidase, rubrerythrins (Rbr) that obtains reducing equivalents from reduced rubredoxin (Rd_red_) and glutathione peroxidase (Gpx) that obtains reducing equivalents from glutathione, were conserved in only *Scalindua* sp.

Based on the comparative genome analysis, it was clearly confirmed that all anammox species commonly possess the genes considered to function for O_2_ reduction (*i.e*., *fdpA* and *fdpF*), O_2_^•-^ reduction (i.e., *nlr*), and H_2_O_2_ reduction (*i.e*., *rbr* and *ccp*) for survival under microaerobic conditions. The Sor-peroxidase dependent O_2_^•-^ and H_2_O_2_ reduction system is much advantageous to anammox bacteria because O_2_ is not generated in these reduction reactions. However, the Sor-peroxidase dependent detoxification system alone may not be sufficient for cell survival under high O_2_ conditions. Intriguingly, only “*Ca*. *Scalindua* sp.” possess the genes for a classical Sod-Cat dependent O_2_^•-^ and H_2_O_2_ detoxification system (*i.e*., *sodA* or *sodB*) and a functional Fdp-rubredoxin operon, which could be responsible for the higher O_2_ tolerance than other freshwater anammox species. The presence of Sod-Cat system on the *Scalindua* genome triggered the experimental investigation of those enzymatic activities.

### Activities of anti-oxidative enzymes in different anammox bacteria

**Fig. 6:**
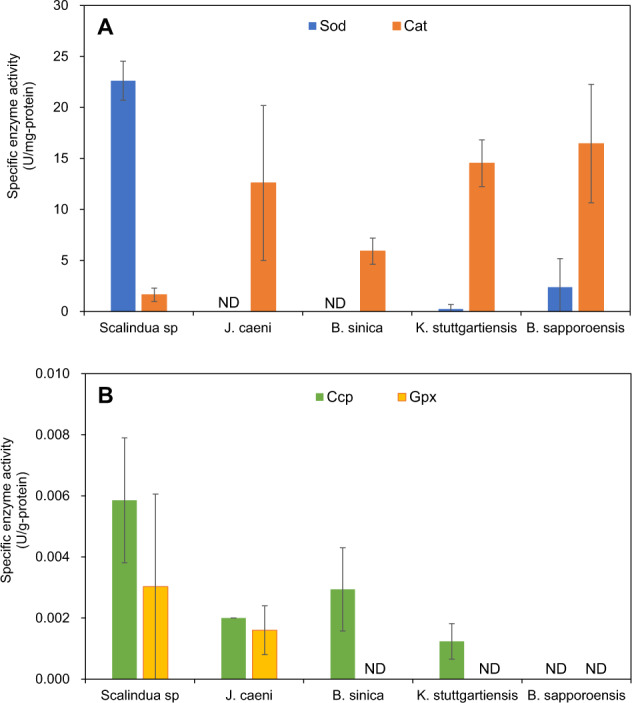
Activities of anti-oxidative enzymes. (**A**: Sod and Cat, **B**: Ccp and Gpx) in cell-free extracts of anammox bacteria harvested from respective anaerobic MBR cultures. Results are presented as the standard error of the mean of at least 6 samples in two independent experiments.

To explain the inter-species difference in O_2_ tolerance, the activities of major anti-oxidative enzymes (Sod, Cat, Ccp, and Gpx) of “*Ca*. Scalindua sp.”, “*Ca*. J. caeni”, “*Ca*. B. sinica”, “*Ca*. B. sapporoensis” and “*Ca*. K. stuttgartiensis” were assayed for their cell-free extracts prepared from biomass collected from respective anaerobic MBR cultures. Only *Scalindua* exhibited high Sod (converts O_2_^•-^ to H_2_O_2_ and O_2_) activity of 22.6 ± 1.9 U/mg-protein with relatively low Cat (converts H_2_O_2_ to O_2_) activity of 1.6 ± 0.7 U/mg-protein (Fig. 6A). This Sod activity level is similar to those of aerobic and facultative bacteria (10.9–49.7 U/mg-protein) [66], but slightly higher than aerotolerant anaerobic bacteria (0.44–19.6 U/mg-protein) and higher than intermediate and extremely oxygen sensitive (obligately) anaerobic bacteria (almost not detected) [67]. In contrast, other four freshwater anammox species possessed very low or virtually no Sod activity with moderately high levels of Cat activity (5.9–16.5 U/mg-protein), which might reflect their lower oxygen tolerance capability. The gene encoding catalase was not identified in the *J. caeni* and *B. sapporoensis* genomes, which may have been a result of the incomplete nature of the genomes. Since the activities of Sod and Cat were highly dependent on medium compositions and culture conditions [68], the effect of salinity on these activities needs to be investigated in the future. Activities of cytochrome *c* peroxidase (Ccp converts H_2_O_2_ to H_2_O) were detected in all species except for “*Ca*. B. sapporoensis” but 3–4 orders of magnitude lower than the Cat activities (Fig. 6B). Activities of glutathione peroxidase (Gpx converts H_2_O_2_ to H_2_O) were barely detected only in “*Ca*. Scalindua sp.” and “*Ca*. J. caeni”.

### Effect of exposed O_2_ concentrations and exposure time on the activities of anti-oxidative enzymes

Cell-free extracts were prepared from anaerobic MBR cultures of “*Ca*. Scalindua sp.*”*, “*Ca*. B. sinica*”*, “*Ca*. J. caeni*”,* and *“Ca*. K. stuttgartiensis*”* were subjected to two patterns of O_2_ exposure: (1) exposed to different O_2_ concentrations (0, 0.7, 1.4 and 2.1% O_2_) for 12 h and (2) exposed to ambient air (21% O_2_) for different periods of time (0, 0.5, 1 and 2 h). High activity levels of Sod were detected in all samples of “*Ca*. Scalindua sp.”, but no significant effects of exposed O_2_ concentrations and duration of O_2_ exposure on the Sod activity levels were observed even after exposure to ambient air for 2 h (Fig. S12 and Fig. S13). The Cat activity in *“Ca*. Scalindua sp.*”* was also remained unchanged for different O_2_ concentrations and durations of O_2_ exposure. Similarly, O_2_ concentrations and O_2_ exposure times did not significantly affect the activity levels of all anti-oxidative enzymes for other three freshwater species. These results revealed that the activities of all anti-oxidative enzymes could be constitutively expressed and active in all anammox bacteria.

The oxygen tolerance is directly related to the ability of the bacteria to reduce (scavenge) O_2_ and to detoxify O_2_^•-^ (namely Sod activity). Thus, Sod activity is a primary important determinant of oxygen tolerance because O_2_^•-^ is more toxic than H_2_O_2_ [66], whereas Cat is the secondary importance since catalase activity showed no clear correlation to oxygen tolerance [66]. “*Ca*. *Scalindua* sp.” possessed significantly higher Sod activity and therefore exhibited higher oxygen tolerance than other freshwater species. It is likely that O_2_^•-^ was primarily detoxified by Sod in “*Ca*. *Scalindua* sp.”, whereas other four freshwater species cannot efficiently detoxify O_2_^•-^ due to lack of Sod activity. The generated H_2_O_2_ could be converted to O_2_ and H_2_O by Cat and/or to H_2_O by peroxidases (Ccp, Gpx, and/or rubrerythrin (Rbr)) (Fig. S7). However, since Ccp and Rbr were active only in the absence of O_2_ with low levels of H_2_O_2_ and also unable to degrade H_2_O_2_ quickly [69, 70], it is not clear if Ccp and Rbr play a vital role in H_2_O_2_ decomposition or not in “*Ca*. *Scalindua* sp.”. Catalase is the most prominent of the stationary-phase induced scavengers and able to degrade H_2_O_2_ more quickly at higher concentrations, whereas rubrerythrins are used to scavenge low levels of H_2_O_2_ [70]. This suggests that “*Ca*. *Scalindua* sp.” most likely employs different peroxidases including Cat depending on H_2_O_2_ concentration [51]. Further study is definitely required to identify which enzyme(s) acts effectively in the H_2_O_2_ detoxification in “*Ca*. *Scalindua* sp.”.

In the presence of oxygen, anammox bacteria reduce O_2_ to form toxic ROS, or reduce to H_2_O by Sor. If bacteria reduce no oxygen and therefore no toxic ROS is generated, resulting in higher oxygen tolerance [66]. “*Ca*. Scalindua sp.” with high Sod activity reduced O_2_ at a relatively slow rate (0.26 µmole O_2_/g-protein/h), whereas “*Ca*. K. stuttgartiensis” that has substantially no Sod activity reduced O_2_ at a more rapid rate (0.53 µmole O_2_/g-protein/h). Although “*Ca*. B. sinica” has no Sod activity, they reduced O_2_ at a relatively slow rate (0.26 µmole O_2_/g-protein/h). These results suggest that “*Ca*. K. stuttgartiensis” generate SOR rapidly but could not detoxify it quickly, resulting in lower O_2_ tolerance and irreversible recovery. It should be also noted that the oxygen reduction rate is an important factor in determining oxygen tolerance.

In conclusion, a marine species, “*Ca*. Scalindua sp.”, exhibited the higher aerotolerance and reversibility than other freshwater anammox species. This is primarily because “*Ca*. Scalindua sp.” possesses the classical Sod-Cat ROS detoxification system in addition to the Sor-peroxidase dependent O_2_ and ROS reduction system. The upper DO limit for “*Ca*. Scalindua sp.” (~51.6 µM) was much higher than the values reported so far (~20 µM). This might suggest that the contribution of anammox to oceanic nitrogen loss could be larger than we have ever thought.

## Supplementary information

Supplemental Information

TableS1

## Data Availability

Anammox bacterial genomes and metagenome-assembled genomes examined in the present study were available in the GenBank and DDBJ database under the following accession number; GCA_900696655.1, GCA_000296795.1, GCA_000786775.1, GCA_000949635.1, GCA_000987375.1, GCA_001723765.1, GCA_001753675.2, GCA_001824485.1, GCA_001828415.1, GCA_001828515.1, GCA_001828545.1, GCA_001828565.1, GCA_001828595.1, GCA_001828605.1, GCA_001828645.1, GCA_001830285.1, GCA_002009475.1, GCA_002050315.1, GCA_002050325.1, GCA_002443295.1, GCA_004282745.1, GCA_005524015.1, GCA_007859995.1, GCA_016187625.1, GCA_016206465.1, GCA_900232105.1, GCF_000949635.1, GCF_001753675.2, GCF_000296795.1, GCF_900232105.1, GCA_004282745.1, BQMK01000001-BQMK01000087, BQMM01000001-BQMM01000139, BQMT01000001-BQMT01000095, BQMU01000001-BQMU01000391, BQMX01000001-BQMX01000120.
