# Supplementary material for: Oxygen tolerance and detoxification mechanisms of highly enriched planktonic anaerobic ammonium-oxidizing (anammox) bacteria
Source: ISME Commun. 2023 May 3;3:45. doi: 10.1038/s43705-023-00251-7 (PMC10156729; doi:10.1038/s43705-023-00251-7)
Supplement: Supplementary file 1 — Supplemental Information [file 43705_2023_251_MOESM1_ESM.docx]

**Supplemental Information**

**Oxygen tolerance and detoxification mechanisms of highly enriched planktonic anaerobic ammonium-oxidizing (anammox) bacteria**

**by**

**Satoshi Okabe^1*^, Shaoyu Ye^1^, Lan Xi^1^, Keishi Nukada^1^, Haozhe Zhang^1^,**

**Kanae Kobayashi^1, 2^, and Mamoru Oshiki^1^**

^1^ Department of Environmental Engineering, Faculty of Engineering,

Hokkaido University. North-13, West-8, Kita-ku, Sapporo Hokkaido, 060-8628, Japan.

2 Super-cutting-edge Grand and Advanced Research (SUGAR) Program,

Japan Agency for Marine-Earth Science and Technology (JAMSTEC),

2-15 Natsushima-cho, Yokosuka city, Kanagawa, 237-0061, JAPAN

*Corresponding author

Satoshi Okabe, Ph.D.

e-mail: [sokabe@eng.hokudai.ac.jp](mailto:sokabe@eng.hokudai.ac.jp)

**
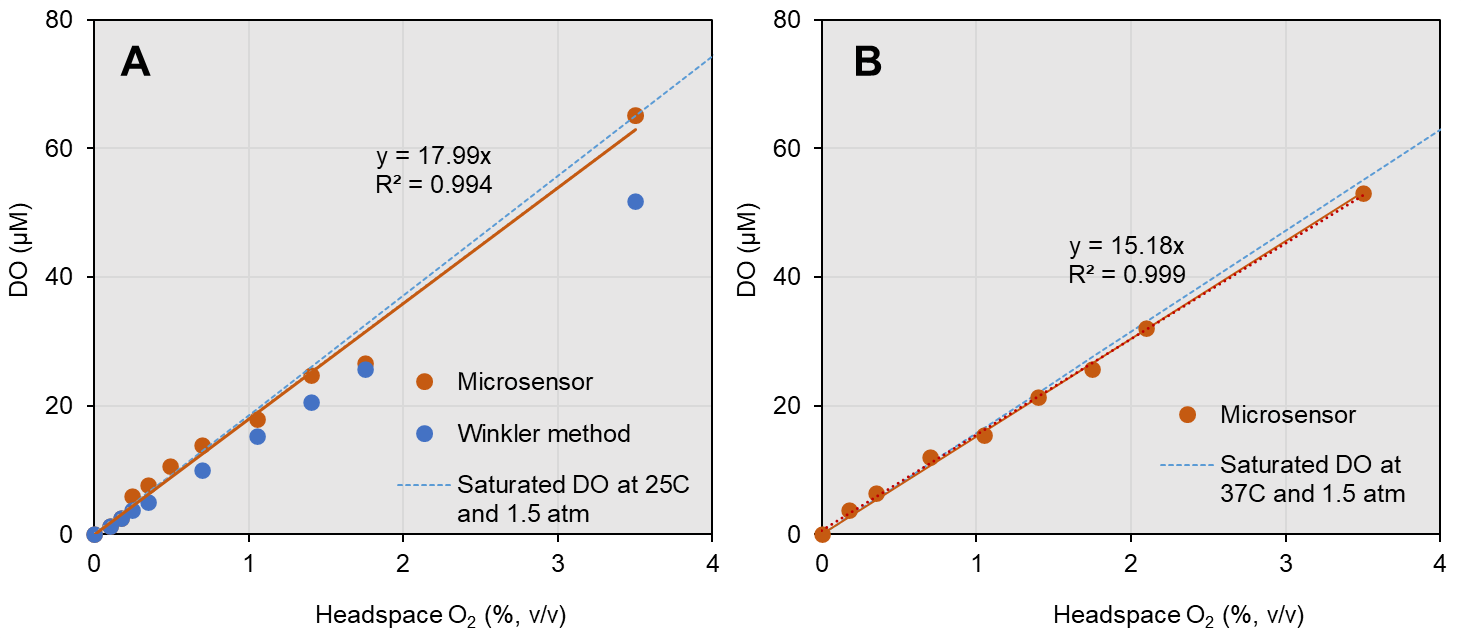
**

**Figure S1. The standard curves of the measured DO concentrations vs. the headspace O_2_ concentrations (%, v/v).** Relationship between the headspace O_2_ concentrations (%, v/v) and dissolved O_2_ (DO) concentrations in the culture medium at 25°C (**A**) and 37°C (**B**). The DO concentrations were measured by a microsensor and by the Winkler method (only for 25°C) were well correlated to the theoretical saturated DO values at 1.5 atm at 25°C and 37°C, respectively.

**
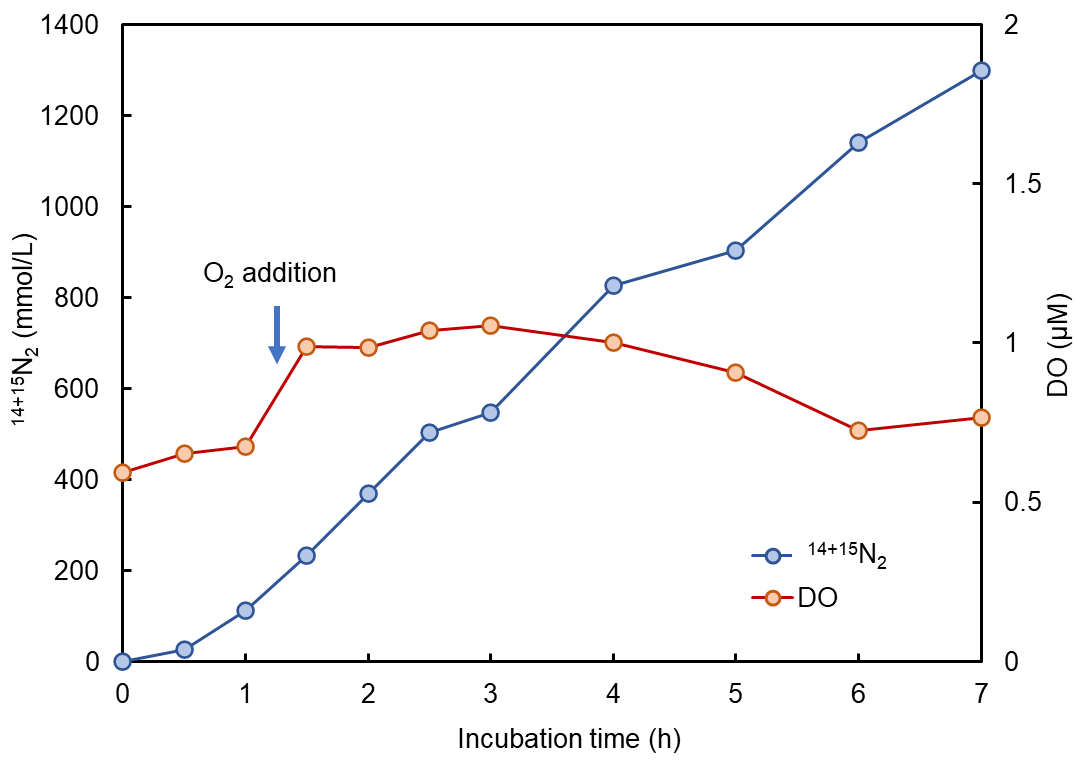
**

**Figure S2. Change in DO concentration over time during an 8-h oxygen inhibition batch experiment.** The Percoll-purified *Scalindua* sp. was cultured by adding ^15^NH_4_^+^ and ^14^NO_2_^-^ at 0 h, and 100 μL of O_2_ gas (100%) was injected into the headspace at 89 min as indicated by an arrow. DO concentration did not change much while ^14+15^N_2_ was steady produced.

**
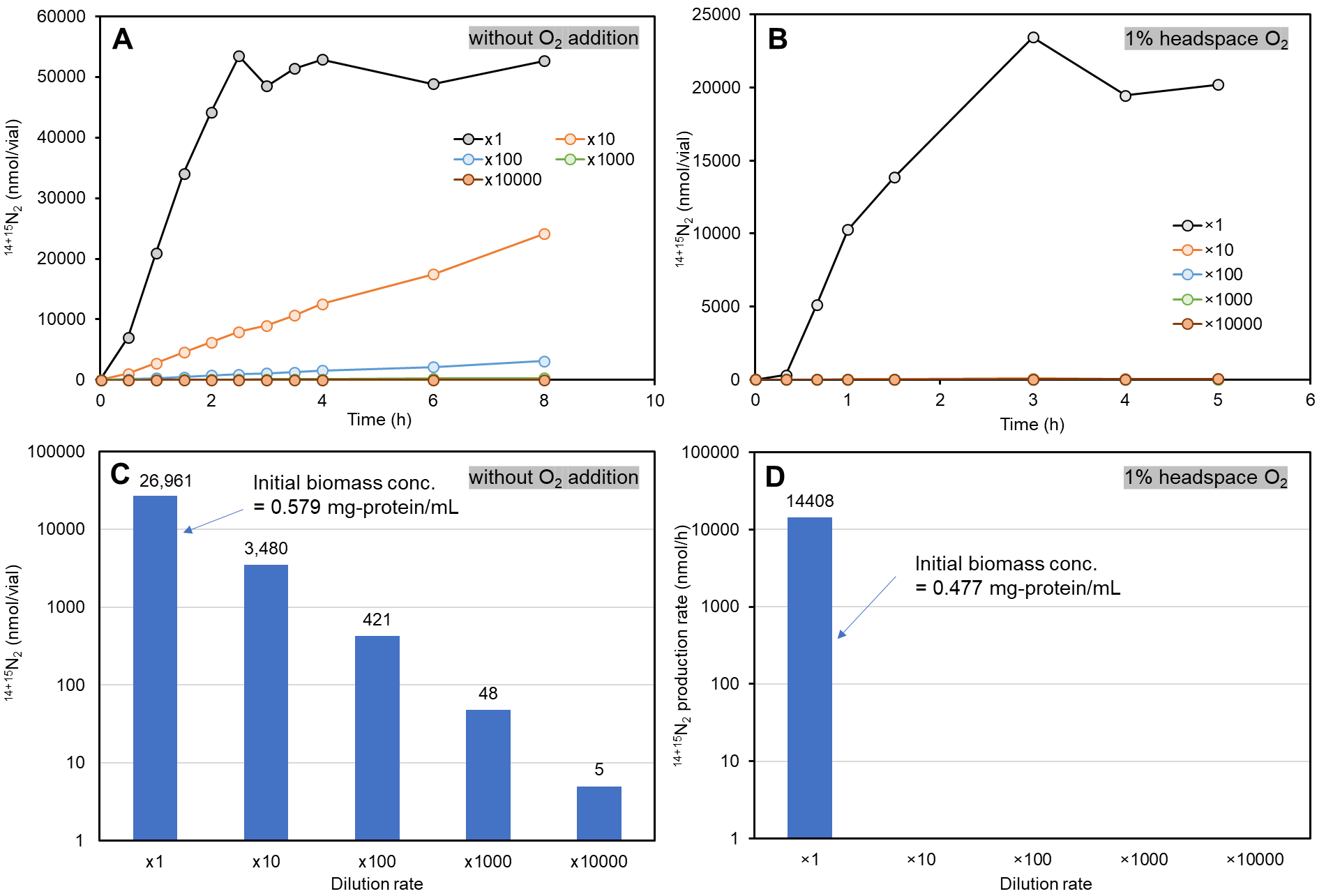
**

**Figure S3.** **Inoculum size-dependent O_2_ tolerance ability of “*Ca*. Scalindua sp.”.** When biomass was diluted tenfold, ^14+15^N_2_ production rate per vial decreased by a factor of 10 down to 10^4^ dilutions without O_2_ % injection (**A** and **C**). However, ^14+15^N_2_ production could not be detected as yet when biomass was diluted tenfold under 1.0% headspace O_2_ (**B** and **D**), showing high susceptibility to oxygen inhibition at low biomass density.


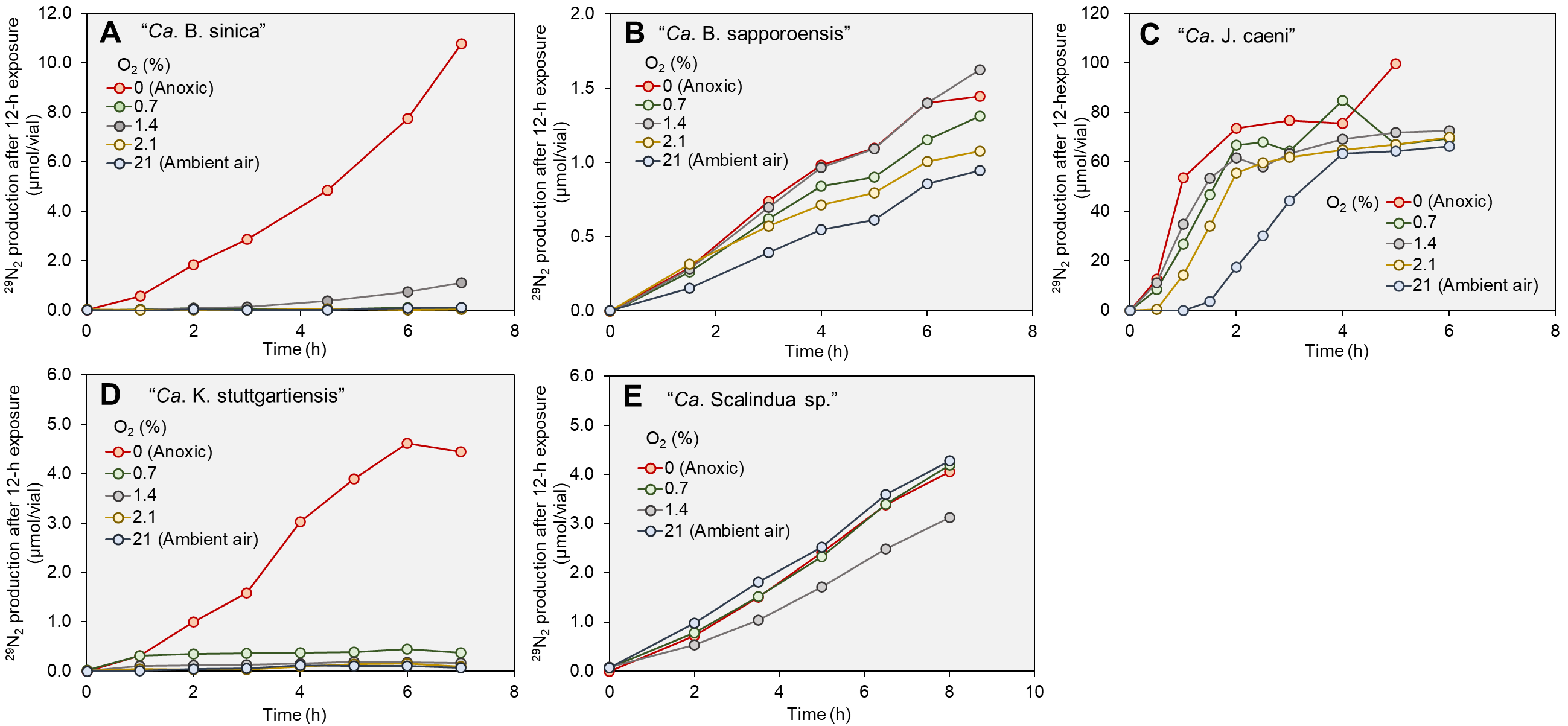


**Figure S4. Immediate recovery of specific anammox activity (SAA) after 12 h-exposure to different headspace O_2_ concentrations.** Typical ^14+15^N_2_ profiles of “*Ca*. B. sinica” (**A**), “*Ca*. B. sapporoensis” (**B**), “*Ca*. J. caeni” (**C)**, “*Ca*. K. stuttgartiensis” (**D**), and “*Ca*. Scalindua sp*.*” (**E**). After exposure to O_2_ concentrations of 0, 0.7, 1.4, 2.1, and 21% (ambient air) for 12 h in the absence of NH_4_^+^ and NO_2_^-^, the culture media were purged with pure Helium gas (>99.9999%) to restore the anaerobic condition, and (^14^NH_4_)_2_SO_4_ (3 mM) and Na^15^NO_2_ (3 mM) were supplemented at 0 h, and then ^14+15^N_2_ gas production was monitored. Anoxic incubations without oxygen exposure (O_2_=0%) were used as anoxic controls. Recovery batch experiments were performed in triplicate, one of which was presented here.


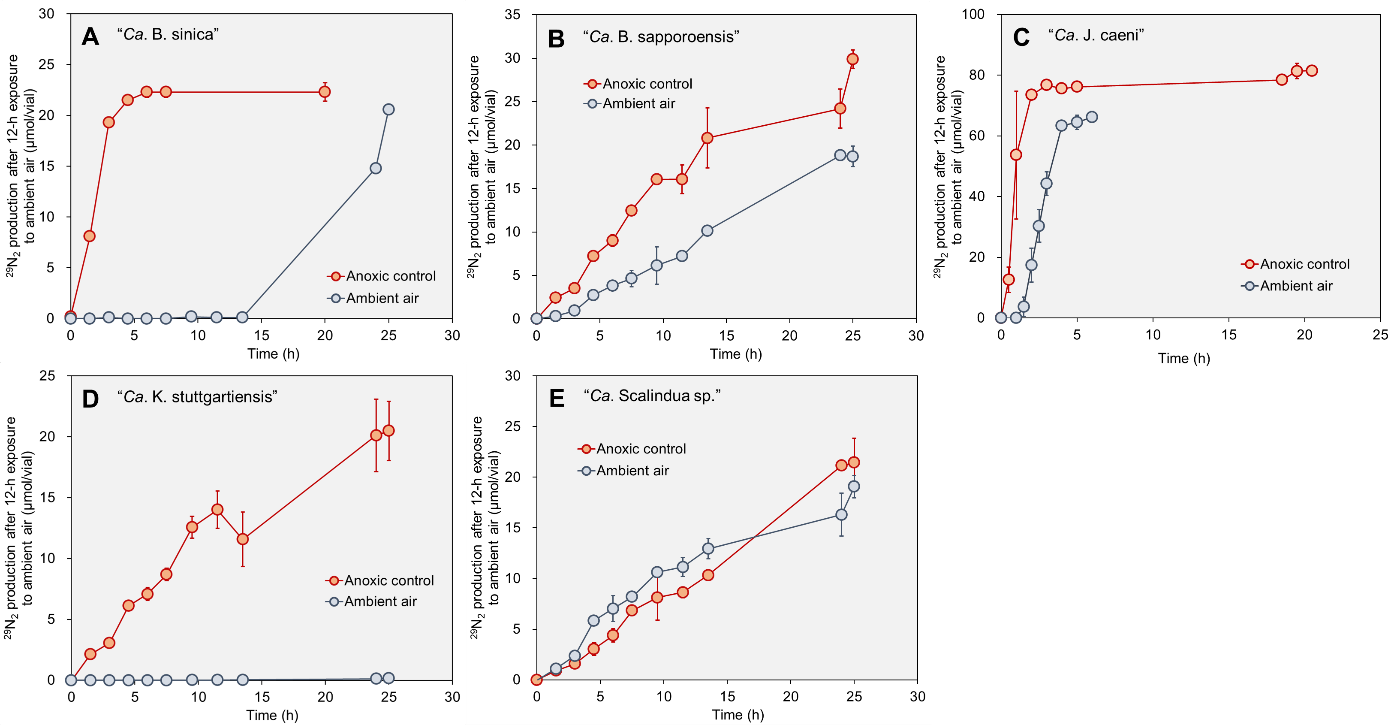


**Figure S5. Recovery of anammox activity after 12-h exposure to ambient air.** Typical ^14+15^N_2_ profiles after restoration of anaerobic conditions from 12-h exposure to ambient air in the absence of NH_4_^+^ and NO_2_^-^ for “*Ca*. B. sinica” (**A**), “*Ca*. B. sapporoensis” (**B**), “*Ca*. J. caeni” (**C)**, “*Ca*. K. stuttgartiensis” (**D**), and “*Ca*. Scalindua sp*.*” (**E**). After exposure to O_2_, the culture media were purged with pure Helium gas (>99.9999%) to restore the anaerobic condition, (NH_4_)_2_SO_4_ (3 mM) and Na^15^NO_2_ (3 mM) were supplemented at 0 h. The error bars represent the standard deviations of duplicate samples. It should be noted that “*Ca.* B. sapporoensis” formed small aggregates, and the biomass of “*Ca.* J. caeni” was about 10 times higher than other species. These could be reasons for almost complete recovery from exposure to ambient air. Recovery batch experiments were performed in triplicate, one of which was presented here.


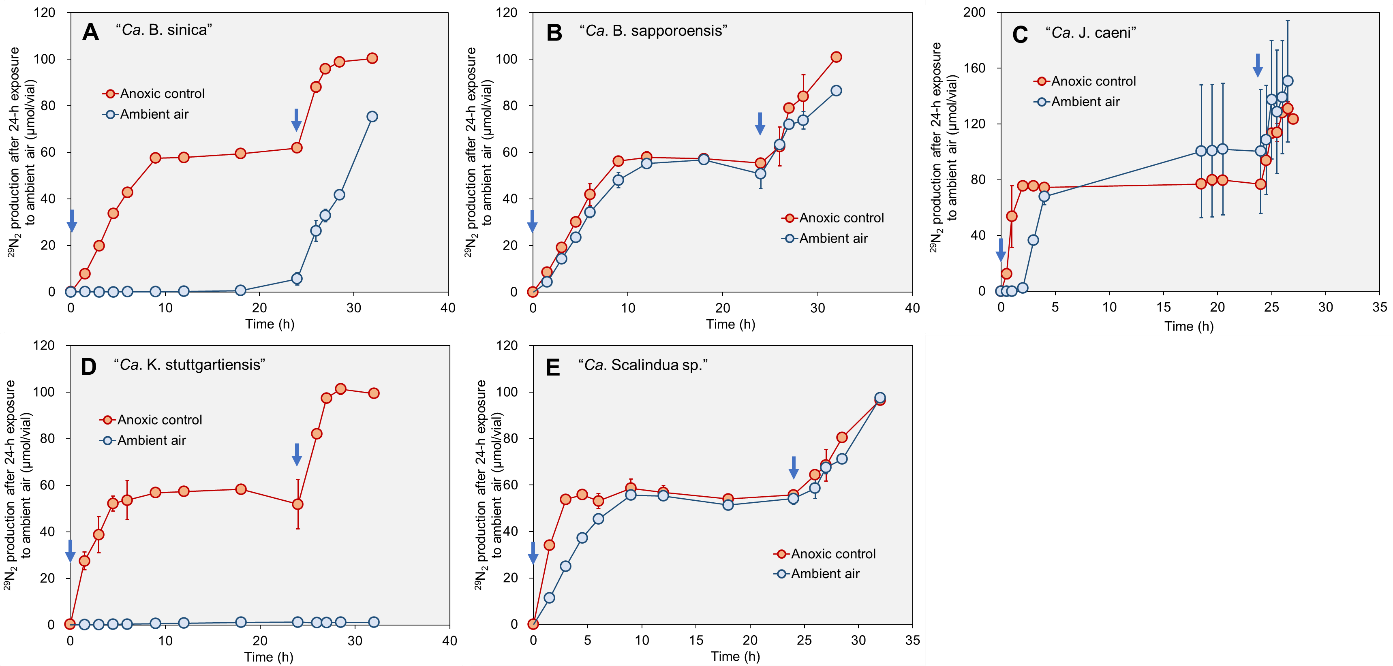


**Figure S6. Recovery of anammox activity after 24-h exposure to ambient air.** Typical ^14+15^N_2_ profiles after exposure to ambient air for 24-h in the absence of NH_4_^+^ and NO_2_^-^ for “*Ca*. B. sinica” (**A**), “*Ca*. B. sapporoensis” (**B**), “*Ca*. J. caeni” (**C)**, “*Ca*. K. stuttgartiensis” (**D**), and “*Ca*. Scalindua sp*.*” (**E**). After exposure to ambient air, the culture media were purged with pure Helium gas (>99.9999%) to restore the anaerobic condition, and (^14^NH_4_)_2_SO_4_ (3 mM) and Na^15^NO_2_ (3 mM) were supplemented at 0 h and 24 h as indicated by arrows. Anoxic incubations without oxygen exposure were used as anoxic controls. The error bars represent the standard deviations of duplicate samples. It should be noted that “*Ca.* B. sapporoensis” formed small aggregates, and the biomass of “*Ca.* J. caeni” was about 10 times higher than other species. These could be reasons for almost complete recovery from exposure to ambient air.


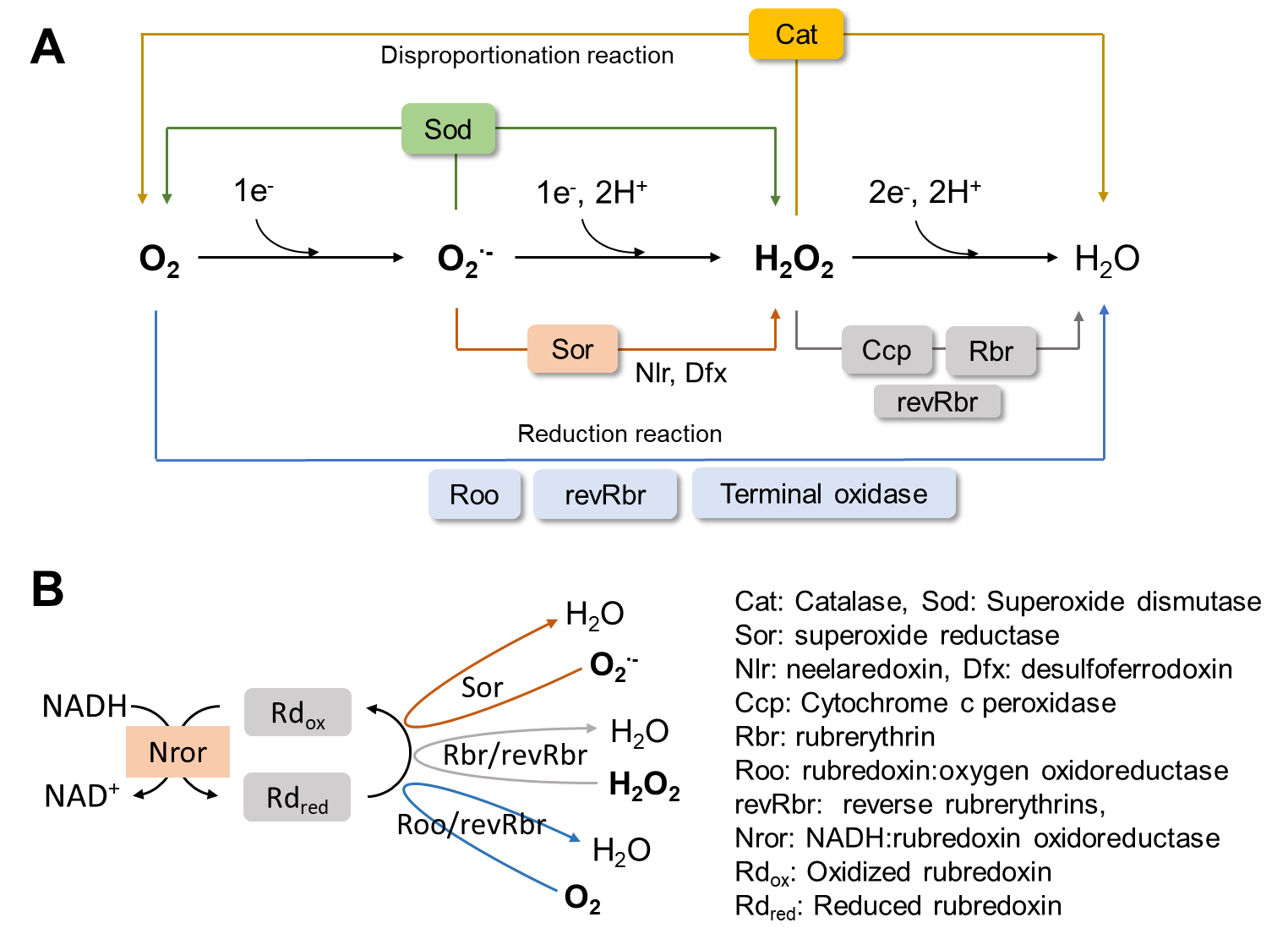


**Figure S7**. **Proposed** **ROS detoxification mechanisms.** (**A**) Proposed oxygen and reactive oxygen species (ROS) detoxification pathways and involved enzymes. (**B**) NADH dependent regeneration of a potential electron donor, rubredoxin (reduced, Rd_red_), for reduction reactions of oxygen and ROS. Enzymes and proteins are hypothetical based on genome sequence analyses.


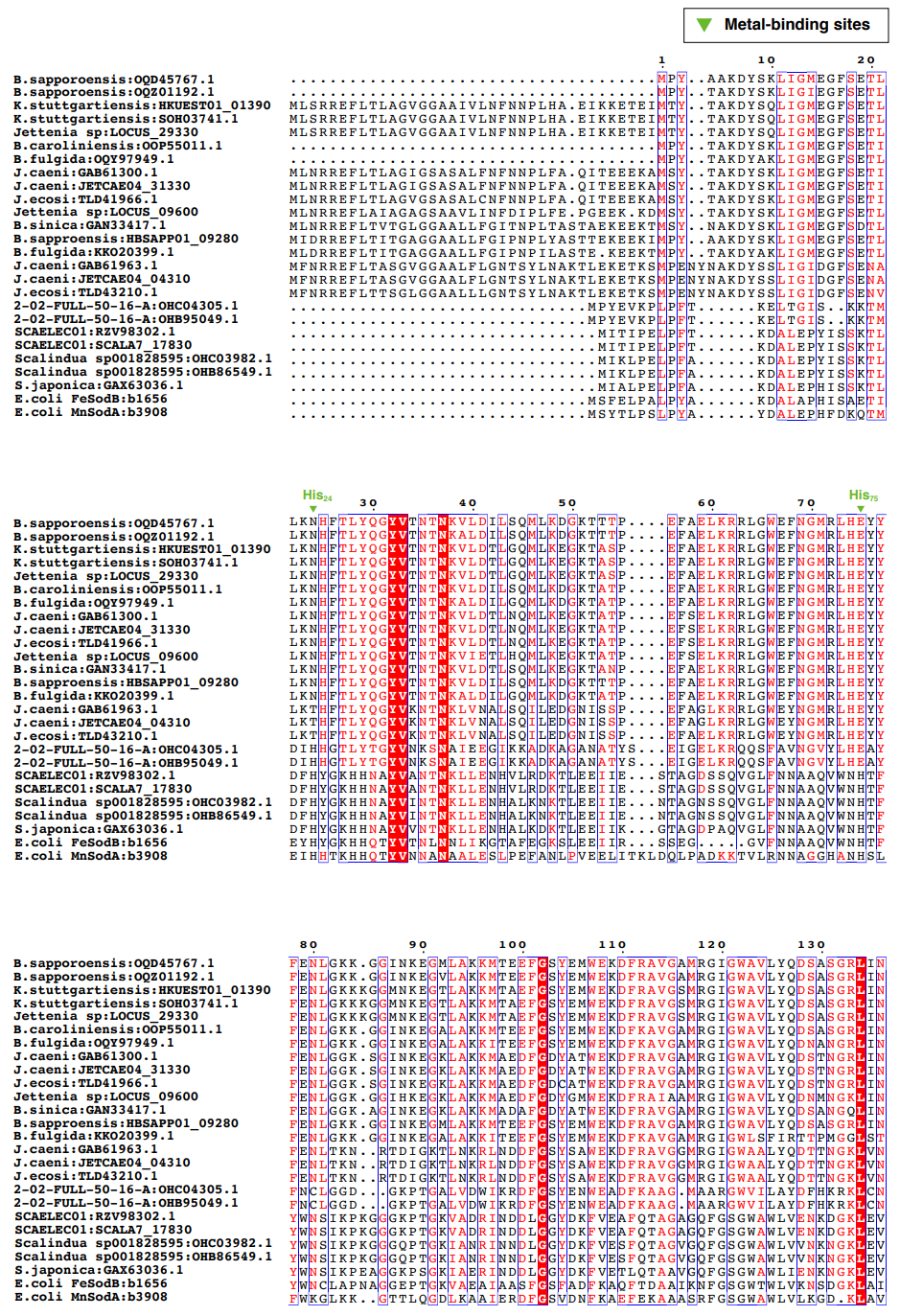


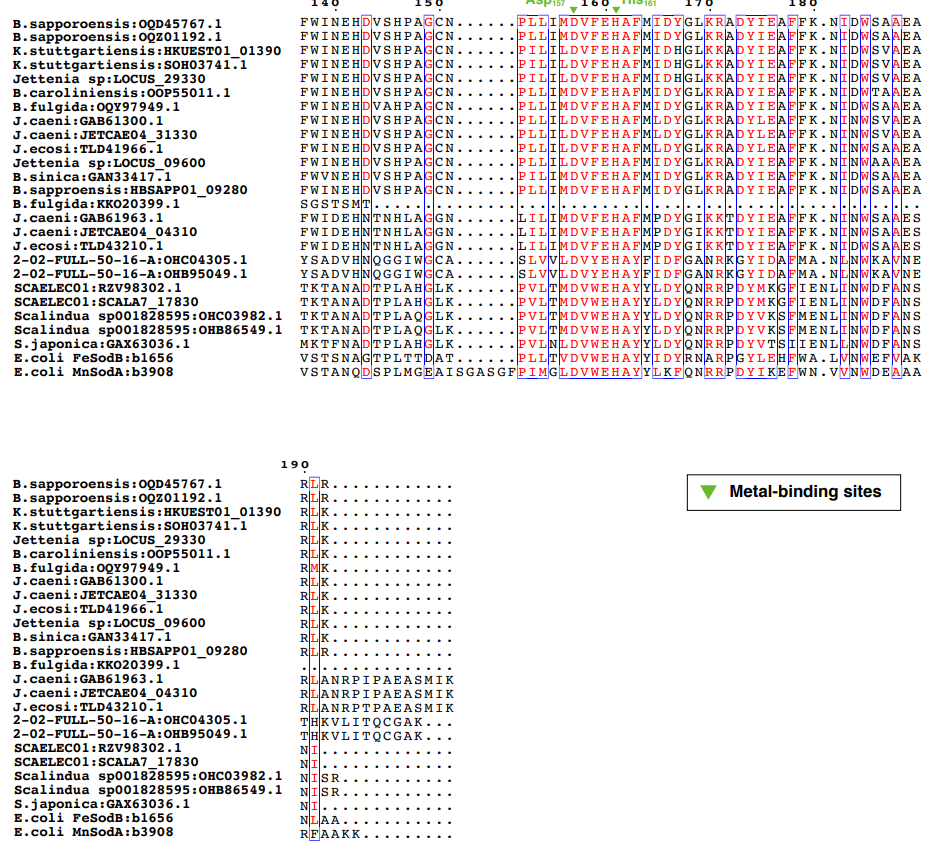


**Figure S8. Sod alignments.** Protein sequences of anammox bacterial Sod were aligned using a ClustalW 1.83. Gap opening and extension penalties in a pairwise alignment were set to 10 and 0.1, respectively. The metal-binding sites found in *E. coli* catalase (PDB ID, 2NYB) (His_24_, His_75_, Asp_157_, and His_161_) were indicated with the triangle symbols.

**
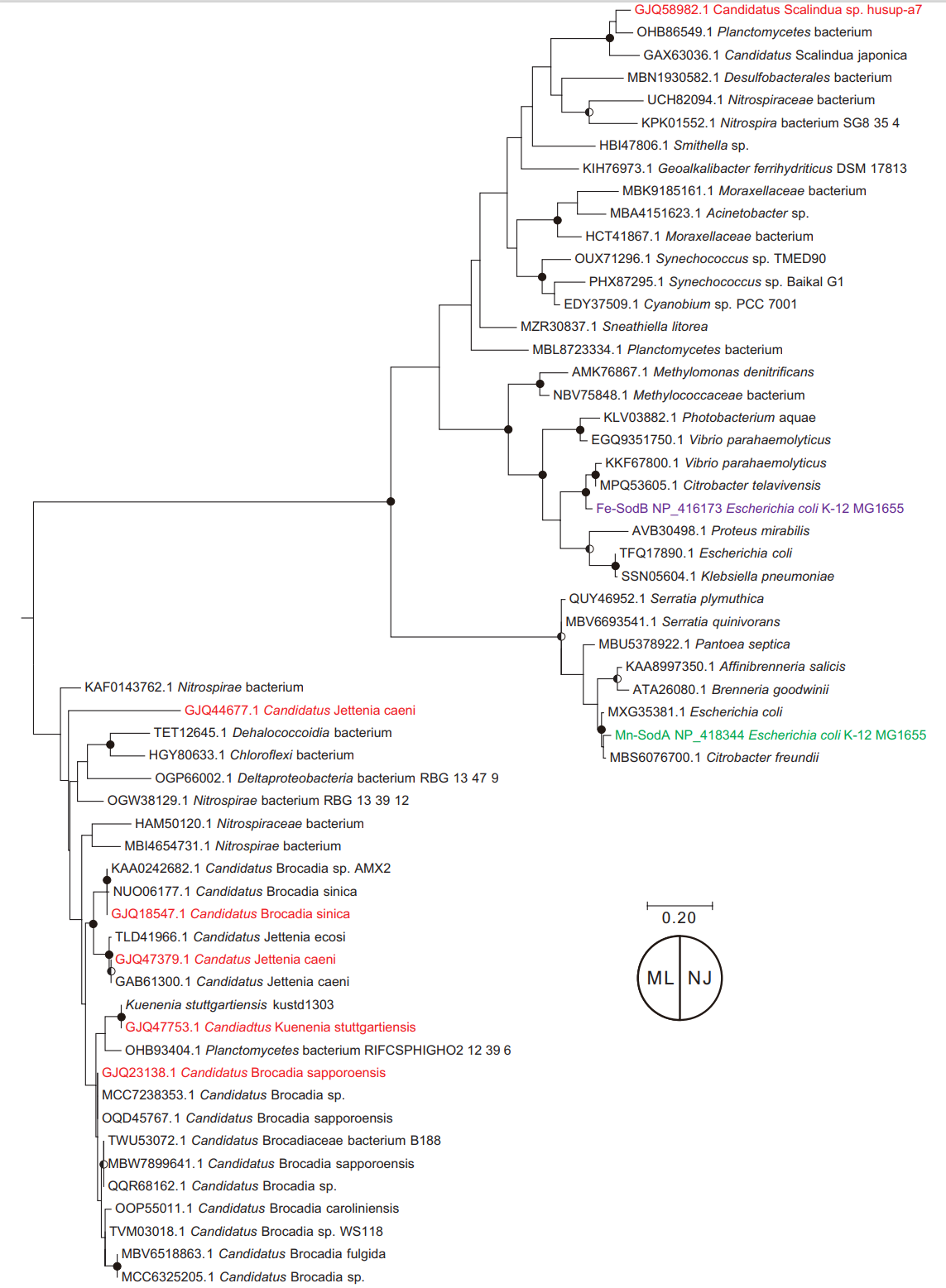
**

**Figure S9. Maximum likelihood (ML) tree showing phylogenetic position of anammox bacterial and *E. coli* super oxide dismutase (Sod).** Branching points that support probability >80% in the bootstrap analysis (based on 200 replicates), estimated using the ML method and the neighbor joining (NJ) method are shown as filled symbol. The scale bar represents 20% sequence divergence. Protein sequence of the Sod of *Geobacter sulfurreducens* PCA (AAR34534.1) was used as the outgroup.


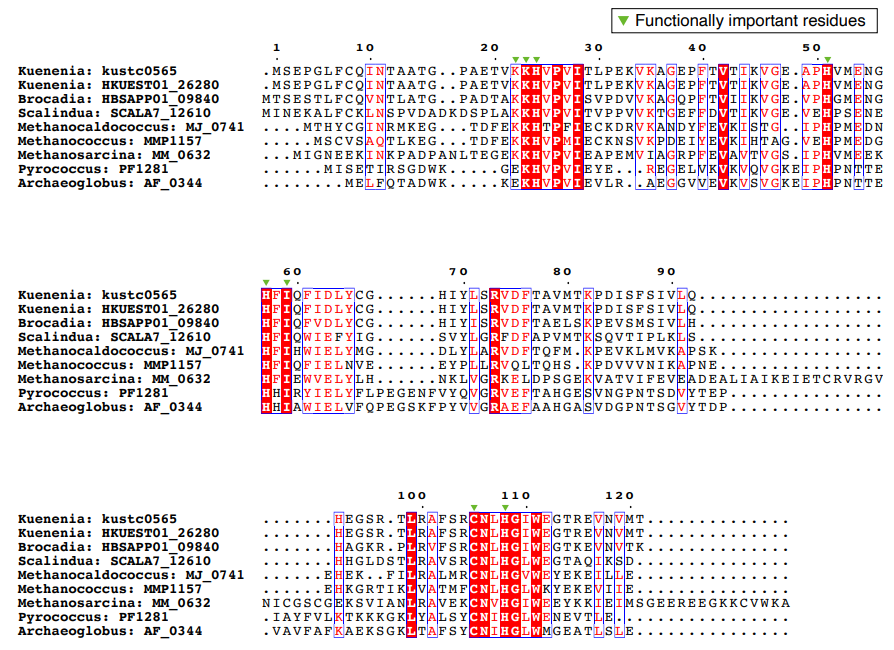


**Figure S10. Sor alignments.** Protein sequences of anammox bacterial Sor were aligned using a ClustalW 1.83. Gap opening and extension penalties in a pairwise alignment were set to 10 and 0.1, respectively. The functionally-important residues of Sor (Pereira A.S. *et al*., 2007, Superoxide reductases. *Eur. J. Inorg. Chem.* **2007**: 2569–2581) were indicated with the triangle symbols.

**Figure S11. Maximum likelihood (ML) tree showing phylogenetic position of anammox bacterial catalase.** Branching points that support probability >80% in the bootstrap analysis (based on 200 replicates), estimated using the ML method and the neighbor joining (NJ) method are shown as filled symbol. The scale bar represents 20% sequence divergence. Protein sequence of the catalase of *Actinomycetia* bacterium (NIA31654.1) was used as the outgroup.

**
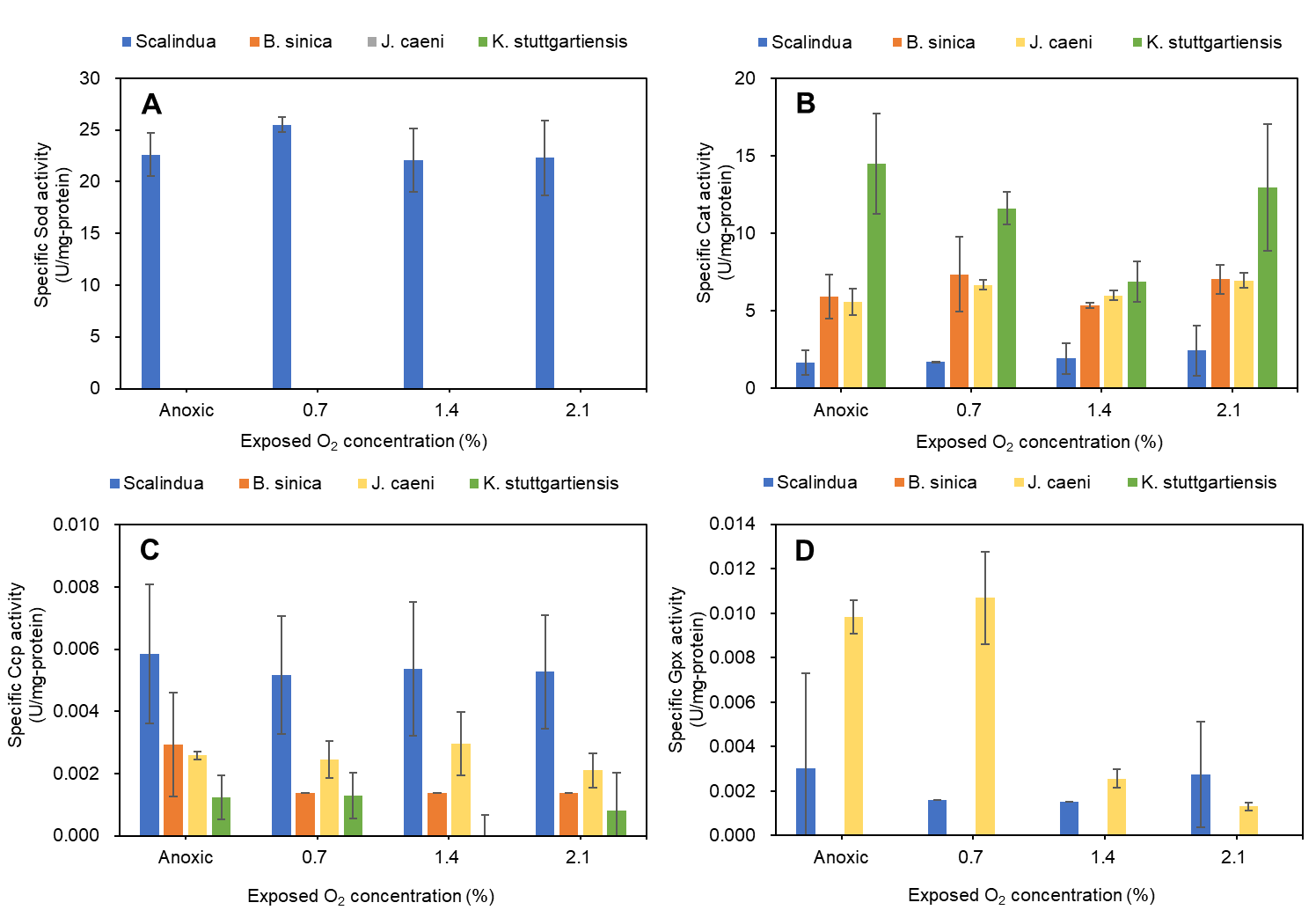
**

**Figure S12. Effect of exposed O_2_ concentrations on anti-oxidative enzymes activities.** Activities of anti-oxidative enzymes (**A**: Sod, **B**: Cat, **C**: Ccp, and **D**: Gpx) in cell-free extracts of anammox bacteria prepared from respective anaerobic MBRs after exposure to different O_2_ concentrations (0 (Anoxic), 0.7, 1.4, and 2.1% O_2_) for 12 h. Results are presented as the mean of at least 6 samples in two independent experiments. The error bars represent the standard deviations. It should be noted that “*Ca*. B. sapporoensis could not be tested because the dominance ratio of MBR enrichment culture was less than 90%.

C

A

**
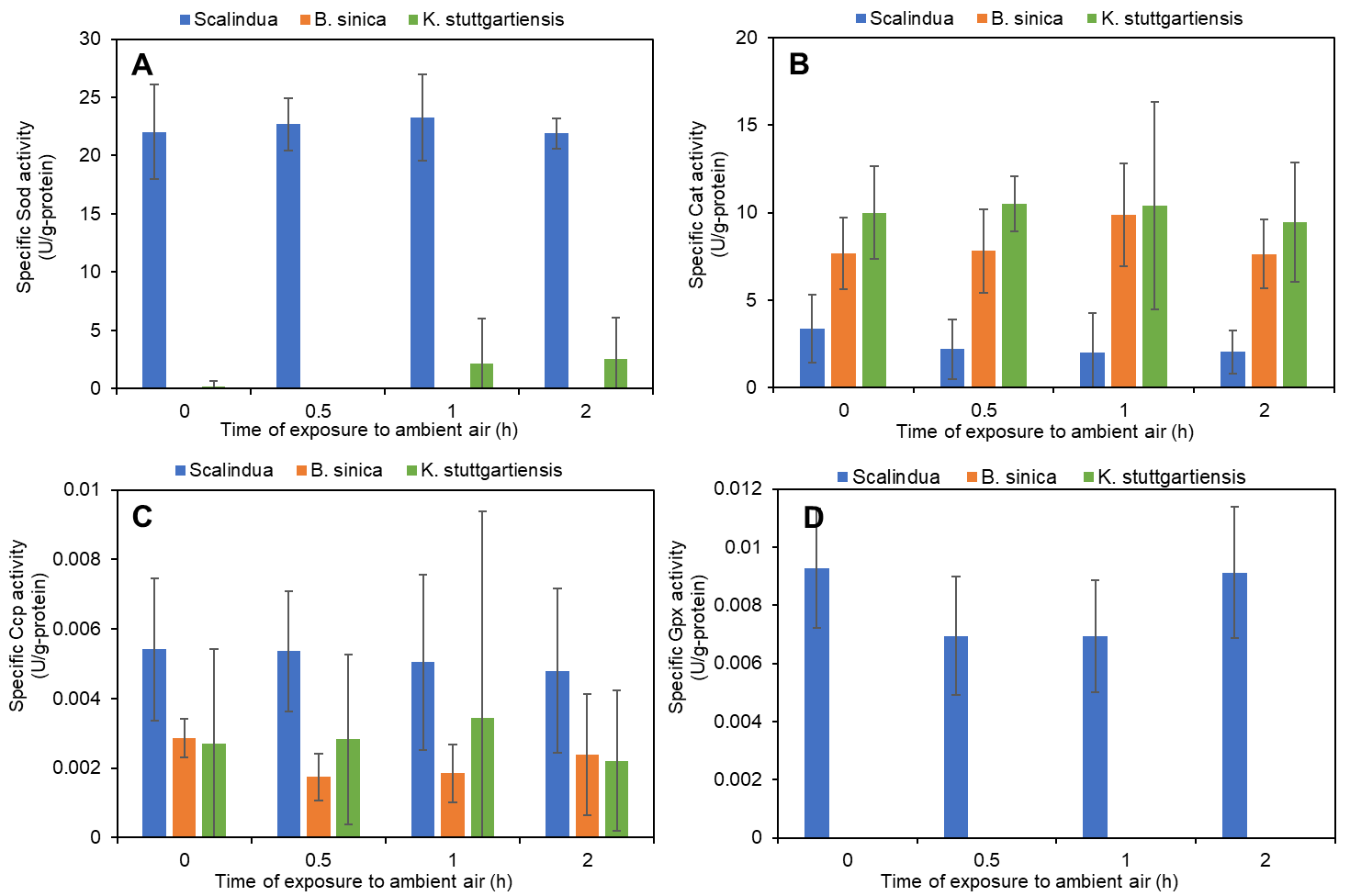
**

**Figure S13. Effect of durations of O_2_ exposures on anti-oxidative enzymes activities.** Activities of anti-oxidative enzymes (**A**: Sod, **B**: Cat, **C**: Ccp, and **D**: Gpx) in cell-free extracts of anammox bacteria prepared from respective anaerobic MBRs after exposure to ambient air for different periods of time (0, 0.5, 1, and 2 h). Results are presented as the mean of at least 6 samples in two independent experiments. The error bars represent the standard deviations. It should be noted that the enzyme activities in “*Ca*. J. caeni” and “*Ca*. B. sapporoensis” could not be measured because the dominance ratios of respective MBR enrichment cultures were less than 90%, respectively.

**Genome sources**

Ali, M., Haroon, M. F., Narita, Y., Zhang, L., Shaw, D. R., Okabe, S. et al. Draft genome sequence of the anaerobic ammonium-oxidizing bacterium “*Candidatus* Brocadia sp. 40”. Genome Announc. 2016; **4**(6): e01377-16.

Awata, T., Tanabe, K., Kindaichi, T., Ozaki, N., Ohashi, A. Influence of temperature and salinity on microbial structure of marine anammox bacteria. Wat Sci Technol. 2012; **66**(5): 958-964.

Frank, J., Lücker, S., Vossen, R. H., Jetten, M. S., Hall, R. J., den Camp, H. J. O. et al. Resolving the complete genome of *Kuenenia stuttgartiensis* from a membrane bioreactor enrichment using single-molecule real-time sequencing. Sci Rep. 2018; **8**(1): 4580.

Oshiki, M., Shinyako-Hata, K., Satoh, H., Okabe, S. Draft genome sequence of an anaerobic ammonium-oxidizing bacterium, “*Candidatus* Brocadia sinica”. Genome Announc. 2015; **3**(2): e00267-15.

Oshiki, M., Mizuto, K., Kimura, Z. I., Kindaichi, T., Satoh, H., Okabe, S. Genetic diversity of marine anaerobic ammonium‐oxidizing bacteria as revealed by genomic and proteomic analyses of “*Candidatus* Scalindua japonica”. Environ Microbiol Rep. 2017; **9**(5): 550-561.

Farr, S. B., Kogoma, T. Oxidative stress responses in *Escherichia coli* and *Salmonella typhimurium*. Microbiol Rev. 1991; **55**: 561–585.

Ferousi, C., Speth, D. R., Reimann, J., den Camp, H. J. O., Allen, J. W., Keltjens, J. T. et al. Identification of the type II cytochrome c maturation pathway in anammox bacteria by comparative genomics. BMC Microbiol. 2013; **13**(1): 265. doi: 10.1186/1471-2180-13-265.

Fridovich, I. Superoxide radical: an endogenous toxicant. Annu Rev Pharmacol Toxicol. 1983; **23**: 239–257.

Imlay, J. A. Linn, S. DNA damage and oxygen radical toxicity. Science. 1988; **240**: 1302–1309.

Katsuwon, J., Anderson, A. J. Response of plant-colonizing pseudomonads to hydrogen peroxide. Appl Environ Microbiol. 1989; **55**(11): 2985-2989.

Kaya, A., Mariotti, M., Gladyshev, V. N. Cytochrome c peroxidase facilitates the beneficial use of H_2_O_2_ in prokaryotes. Proc. Natl. Acad. Sci. 2017; **114** (33): 8678-8680.

Lam, P., Lavik, G., Jensen, M.M., van de Vossenberg, J., Schmid, M., Woebken, D., et al. Revising the nitrogen cycle in the Peruvian oxygen minimum zone. Proc Natl Acad Sci USA. 2009; **106**: 4752–4757.

Lowry, O. H., Rosebrough, N. J., Farr, A. L., Randall, R. J. Protein measurement with the Folin phenol reagent. J Biol Chem. 1951; **193**: 265-275.

Martine, R., Carine, M., José, R. Comparative study of the enzymatic defense systems against oxygen-derived free radicals: The key role of glutathione peroxidase, Free Radical Biology and Medicine, Volume 3, Issue 1. 1987; doi: 10.1016/0891-5849(87)90032-3

Mishra, S., Imaly, J. Why do bacteria use so many enzymes to scavenge hydrogen peroxide? Arch Biochem Biophys. 2012; **525**: 145-160.

Molivian, H. R., Goldman, A., Phipps C. J., Kohandel M., et al. Drug-induced reactive oxygen species (ROS) rely on cell membrane properties to exert anticancer effects. Sci Rep. 2016; **6**: 27439. doi: 10.1038/srep27439.

Revsbech, N. P., Larsen, L. H., Gundersen, J., Dalsgaard, T., Ulloa, O., Thamdrup, B. Determination of ultra‐low oxygen concentrations in oxygen minimum zones by the STOX sensor. Limnol Oceanogr Methods. 2009; **7**(5): 371-381.

Rodríguez, E., Peirotén, Á., Landete, J. M., Medina, M., Arqués, J. L. Gut catalase-positive bacteria cross-protect adjacent *Bifidobacteria* from oxidative stress. Microbes Environ. 2015; **30**: 270–272.

Van de Graaf, A. A., de Bruijn, P., Robertson, L. A., Jetten, M. S., Kuenen, J. G. Autotrophic growth of anaerobic ammonium-oxidizing micro-organisms in a fluidized bed reactor. Microbiol. 1996; **142**(8): 2187-2196.

Winterbourn, C. C., Hawkins, R. E., Brian, M., Carrell, R. W., The estimation of red cell superoxide dismutase activity. J Lab Clin Med. 1975; **85**(2): 337-341.

Yan, Y., Wang, Y., Wang, W., Zhou, S., Wang, J., Guo, J. Comparison of short-term dosing ferrous ion and nanoscale zero-valent iron for rapid recovery of anammox activity from dissolved oxygen inhibition. Water Res. 2019; **153**: 284-294.
